# Heterogeneity in enterotoxigenic *Escherichia coli* and shigella infections in children under 5 years of age from 11 African countries: a subnational approach quantifying risk, mortality, morbidity, and stunting

**DOI:** 10.1016/S2214-109X(19)30456-5

**Published:** 2019-11-14

**Authors:** Karoun H Bagamian, John D Anderson, Farzana Muhib, Oliver Cumming, Lindsey A Laytner, Thomas F Wierzba, Richard Rheingans

**Affiliations:** a Department of Environmental & Global Health, University of Florida, FL, USA; b Bagamian Scientific Consulting, Gainesville, FL, USA; c Goodnight Family Department of Sustainable Development, Appalachian State University, Boone, NC, USA; d PATH, Washington, DC, USA; e Department of Disease Control, London School of Hygiene & Tropical Medicine, London, UK; f Department of Internal Medicine, Section on Infectious Diseases, Wake Forest School of Medicine, Winston Salem, NC, USA

## Abstract

**Background:**

Diarrhoea, a global cause of child mortality and morbidity, is linked to adverse consequences including childhood stunting and death from other diseases. Few studies explore how diarrhoeal mortality varies subnationally, especially by cause, which is important for targeting investments. Even fewer examine indirect effects of diarrhoeal morbidity on child mortality. We estimated the subnational distribution of mortality, morbidity, and childhood stunting attributable to enterotoxigenic *Escherichia coli* (ETEC) and shigella infection in children younger than 5 years from 11 eastern and central African countries. These pathogens are leading causes of diarrhoea in young children and have been linked to increased childhood stunting.

**Methods:**

We combined proxy indicators of morbidity and mortality risk from the most recent Demographic and Health Surveys with published relative risks to estimate the potential distribution of diarrhoeal disease risk. To estimate subnational burden, we used country-specific or WHO region-specific morbidity and mortality estimates and distributed them subnationally by three indices that integrate relevant individual characteristics (ie, underweight, probability of receiving oral rehydration treatment of diarrhoea, and receiving vitamin A supplementation) and household characteristics (ie, type of drinking water and sanitation facilities).

**Findings:**

Characterising ETEC and shigella subnational estimates of indirect morbidity (infection-attributable stunting) and indirect mortality (stunting-related deaths from other infectious diseases) identified high-risk areas that could be missed by traditional metrics. Burundi and Democratic Republic of the Congo had the highest ETEC-associated and shigella-associated mortality and stunting rates. Mozambique, Democratic Republic of the Congo, and Zimbabwe had the greatest subnational heterogeneity in most ETEC and shigella mortality measures. Inclusion of indirect ETEC and shigella mortality in burden estimates resulted in a 20–30% increase in total ETEC and shigella mortality rates in some subnational areas.

**Interpretation:**

Understanding the indirect mortality and morbidity of diarrhoeal pathogens on a subnational level will strengthen disease control strategies and could have important implications for the relative impact and cost-effectiveness of new enteric vaccines. Because our methods rely on publicly available data, they could be employed for national planning.

**Funding:**

Bill & Melinda Gates Foundation.

## Introduction

Diarrhoea remains the second leading cause of global childhood mortality, despite recent estimates suggesting a 44% decline in global diarrhoea mortality from 2000 to 2015.^1^ However, morbidity estimates show a modest decrease from 1·9 million to 1·7 million episodes for children younger than 5 years of age in the past 20 years.^2^ The high incidence of diarrhoeal morbidity is concerning because studies have shown that repeated non-fatal diarrhoeal episodes might increase childhood stunting,^3^, ^4^, ^5^ which increases risk of mortality from other infectious diseases. Including these consequences in diarrhoeal mortality estimates provides a more complete picture of diarrhoea's public health impact. Recent studies that included the impact of growth impairment in burden of diarrhoeal disease estimates in children have reported increases ranging from 24% to 153%.^6^, ^7^, ^8^

Diarrhoeal mortality and morbidity varies within and between countries.^1^, ^9^ In many cases, this variation is caused by underlying patterns of poverty, insufficient access to essential services, and diverse living environments. Describing the heterogeneity in disease risk and burden has important implications for the effectiveness of disease interventions. Studies of infectious disease incidence have shown the importance of geographical differences in disease risk and burden when determining the impact and cost-effectiveness of intervention strategies.^10^, ^11^ Although many studies investigate the importance of heterogeneity in risk of malaria^12^ and measles,^13^ few studies explore subnational diarrhoeal heterogeneity.^11^ Unlike malaria, no agreed-upon metric exists for diarrhoea to determine spatial heterogeneity. Heterogeneity in diarrhoeal risk and mortality burden can be further complicated by pathogen-specific risks. When considering specific enteric pathogens or diarrhoeal mortality more broadly, very little information exists on differences within countries. Studies that evaluate diarrhoea within countries mostly focus on incidence or mortality patterns within one^14^ or a few countries^9^, ^15^ and have not included indirect morbidity and mortality in their estimates.^9^, ^11^

**Research in context**

**Evidence before this study**

Diarrhoea remains a leading cause of childhood mortality, but only recently have large-scale studies produced estimates of the aetiology of diarrhoeal disease in young children living in low-income and middle-income countries. Enterotoxigenic *Escherichia coli* (ETEC) and shigella are leading causes of diarrhoeal morbidity and mortality in children from low-income and middle-income countries and are linked to childhood stunting. The Global Burden of Diseases, Injuries, and Risk Factors Study has provided some cause-specific estimates of diarrhoeal burden, but these estimates are currently only available at national or global regional levels. Additionally, although a few studies have begun to include the indirect effects of diarrhoeal disease (eg, increased stunting leading to increased vulnerability to other infections and mortality from these infections) in their burden estimates, none of these studies include within-country estimates of ETEC and shigella burden. Another study has assessed overall diarrhoeal burden and severity at a subnational level across Africa, but these estimates were not cause specific. We searched the literature and found no studies that included mortality and morbidity burden estimates for ETEC and shigella at a subnational scale or that estimated ETEC and shigella burden including indirect mortality from ETEC-attributable and shigella-attributable stunting. We searched PubMed from inception to Feb 9, 2019, without language or location restrictions. We used two search strings: one for the diarrhoeal disease aetiology in children younger than 5 years and another on diarrhoea and stunting. For diarrhoeal aetiology in children younger than 5 years, we found 68 publications with the search string (“ETEC” OR “enterotoxigenic *E coli”* OR “enterotoxigenic” OR “shigella”) AND (“children under five” OR “children under 5” OR “children <5”) AND (“morbidity” OR “illness” OR “stunting” OR “episodes” OR “events” OR “cases”) AND (“cause” OR “etiology” OR “aetiology”). For diarrhoea and stunting, we found 282 publications with the search string (“diarrhea” OR “diarrea” OR “diarrhoea”) AND (“stunting” OR “height for age” OR “height” OR “Z score”) AND (“child*” OR “children under five” OR “children under 5” OR “children <5”) AND (“outcomes” OR “changes”).

**Added value of this study**

We produced subnational estimates of ETEC and shigella morbidity, mortality, and stunting, as well as the indirect burden of deaths from other infectious diseases attributed to ETEC and shigella stunting, for children living in 11 eastern and central African countries. We integrated our previously published approach with methods for distributing burden estimates across subpopulations. Our study provides a more complete picture of diarrhoeal burden by including acute and potential outcomes of ETEC or shigella episodes at a finer spatial scale than previously published.

**Implications of all the available evidence**

Our analysis showed that within an area of the world with a high burden of diarrhoeal disease, there was considerable variation in ETEC and shigella burden. Subnational estimates revealed large variation in burden and identified susceptible populations of children with high disease burden, indicating the importance of within-country analysis. Assessing cause-specific estimates at the level at which policy makers can more effectively target interventions and investments is crucial to better reach the most vulnerable populations of children.

Recent multisite longitudinal studies in low-income countries have found that enterotoxigenic *Escherichia coli* (ETEC) and shigella are leading causes of moderate-to-severe diarrhoea for children^16^, ^17^ and diarrhoeal episodes attributed to these pathogens are associated with decreased linear growth in children younger than 2 years.^18^ Recent estimates show that several countries in eastern and central Africa have high ETEC and shigella burden,^17^, ^19^ with children younger than 5 years from this region having the highest ETEC mortality rates globally.^19^ Therefore, we chose to explore the subnational patterns of ETEC and shigella morbidity and total mortality in this region. Here, we estimated the spatial distribution of diarrhoeal risk and ETEC and shigella direct morbidity and mortality, stunting cases attributable to ETEC and shigella, and deaths from other infectious diseases due to ETEC-attributable and shigella-attributable stunting in children across 11 countries in eastern and central sub-Saharan Africa.

## Methods

### Study overview and data sources

**Figure 1:**
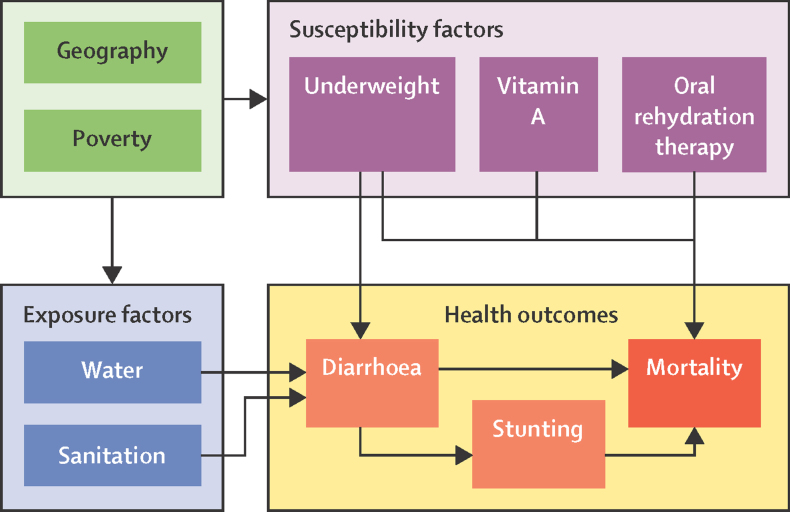
Conceptual framework of the diarrhoeal mortality risk model

We developed disease burden models to estimate ETEC-related and shigella-related mortality and morbidity for children younger than 5 years at the subnational level. We developed an index to score each child on the basis of specific weighted relative risks (RRs) of diarrhoeal mortality drawn from published literature (appendix pp 9–10). The risk index is composed of the RR related to the child's exposure and susceptibility to diarrhoeal diseases (figure 1).^20^ We integrated recent methods for estimating ETEC and shigella mortality, attributable stunting, and related mortality from other diseases^8^ and distributed our rate estimates subnationally using adjusted indices and rates.

To populate our models, we used child-level data from the 2011–17 Demographic and Health Survey (DHS)^21^ for 11 countries in eastern and central sub-Saharan Africa (appendix p 5). Countries in this analysis are contiguous and had recent DHS data available.^21^ All estimates derived from DHS data were calculated using appropriate survey weights. We used Malaria Atlas Project administrative unit level 1 child population estimates,^22^ aggregated into subnational units (referred to here as provinces), as appropriate per country (table 1).

**Table 1:** List of countries from east and central Africa included in the analysis

	**Survey year**	**DHS phase**	**Subnational unit of measure**
Burundi	2016–17	DHS-VII	Administrative unit 1 (18 provinces)
Democratic Republic of the Congo	2013–14	DHS-VI	Administrative unit 1 (by 11 regions based on former provinces and includes one city, Kinshasa)
Ethiopia	2016	DHS-VII	Administrative unit 1 (by nine regional states and two chartered cities)
Kenya	2014	DHS-VII	Administrative unit 1 (by eight regions based on former provinces)
Malawi	2015–16	DHS-VII	Administrative unit 1 (by three regions)
Mozambique	2011	DHS-VI	Administrative unit 1 (by ten provinces and one chartered city)
Rwanda	2014–15	DHS-VII	DHS-determined five regional groupings of administrative 1 areas
Tanzania	2015–16	DHS-VII	DHS-determined eight regional groupings of administrative 1 areas
Uganda	2016	DHS-VII	15 subregional groupings of districts
Zambia	2013–14	DHS-VI	Administrative unit 1 (by ten provinces)
Zimbabwe	2015	DHS-VII	Administrative unit 1 (by ten provinces)

DHS=Demographic and Health Survey.

### Diarrhoeal mortality risk index

We calculated a diarrhoeal mortality risk index *R*_i_ for each child *i* (equation 1.1) that was the product of an exposure index *E*_i_ (equation 1.2) and a susceptibility index *S*_i_ (equation 1.3). The exposure index is composed of the combined RR associated with *m* household-level exposure factors and the susceptibility index is composed of the RRs of *n* child-level susceptibility factors (figure 1).

(1.1) $$R_{i}=E_{i}.S_{i}$$

(1.2) $$E_{i}=\prod_{e=1}^{m}{\left(RR_{ej}.e_{j}\right)}_{i}$$

(1.3) $$S_{i}=\prod_{s=1}^{n}{\left(RR_{s,k}.s_{k}\right)}_{i}$$

The three index scores were calculated for each child younger than 5 years using data from the DHS.^21^ We assumed that factors contributing to increased mortality burden were equivalent for ETEC and shigella because the necessary information to differentiate between the two is not available.

We used previously published RR estimates^23^ to assign a combined score *E*_i_ that assumed child exposure to diarrhoeal mortality risk was determined by two factors (thus, m = 2): the type of drinking water and the sanitation facilities used by their household (equation 1.2).^20^ The exposure score was the product of RRs associated with *j*th level of each exposure factor *e*, where each child could only be categorised into one level of each factor (appendix p 9). RR values were associated with three categories of drinking water sources (unimproved, off-plot improved, or on-plot improved) and with three categories of sanitation facilities (unimproved or no facility; improved and unshared sanitation on premises without a sewer connection; and improved and unshared sanitation on premises with a sewer connection) for a total of nine possible water and sanitation scenarios assigned to each individual child (appendix p 10).

We defined child susceptibility using three risk factors (thus, *n*=3) known to influence diarrhoeal mortality: child undernutrition defined by categories by weight for age, probability of receiving oral rehydration treatment of diarrhoea, and receiving vitamin A supplementation (equation 1.3; figure 1; appendix pp 2–3). The susceptibility score *S*_i_ was the product of the RRs associated with the *k*th level of each susceptibility risk factor *s* for each child *i*, who could only be categorised into one level of each factor (equation 1.3; appendix p 9). *S*_i_ was designed to be proportional to the excess risk associated with all three factors.

### ETEC and shigella burden

We estimated national overall diarrhoeal mortality rates using the midpoint of 2016 WHO Maternal Child Epidemiology Estimation^24^ and Global Burden of Diseases, Injuries, and Risk Factors Study (GBD)^25^ diarrhoeal mortality estimates, adjusted for countries that introduced the rotavirus vaccine.^8^ We calculated the national ETEC and shigella mortality rates for each country *c* by multiplying national diarrhoeal mortality rates *D*_c_ with culture-based aetiological fractions^26^ adjusted with molecular estimates *x*_p_ for each pathogen *p* (appendix pp 7, 11).^16^ We generated provincial ETEC and shigella mortality rates *d*_s,p_ by multiplying national mortality rates by the quotient from dividing the mean diarrhoeal mortality risk index score *R*_s_ for each province *s* by the national mean risk score *R*_c_ for the corresponding country:

$$d_{s,p}=(D_{c}.x_{p}).\frac{R_{s}}{R_{c}}$$

This ensured that the average of provincial mortality rates *d*_s_ remained equal to the national mortality estimates.

We estimated the number of national ETEC and shigella moderate-to-severe diarrhoeal episodes by multiplying the WHO regional diarrhoeal incidence, *M*_c_ (currently 3·3 episodes per child per year),^27^ by aetiological fractions *x* for each pathogen *p*. On the basis of prior assumptions on health-care use^28^ and estimates from the Global Enteric Multicenter study,^17^ we assumed that 10% of diarrhoeal episodes were considered moderate to severe.^8^ We distributed ETEC or shigella episodes across provinces by multiplying national rates of moderate-to-severe diarrhoeal episodes by the quotient from dividing the mean provincial exposure score *E*_s_ by the mean exposure score *E*_c_ for the corresponding country:

$$m_{s,p}=(M_{c}.x_{p}).\frac{E_{s}}{E_{c}}$$

Using previous methodology^8^ and population shifts in height-for-age Z scores due to ETEC and shigella moderate-to-severe diarrhoeal episodes reported by the Global Enteric Multicenter study,^17^ we modelled the portion of childhood stunting attributed to ETEC and shigella infections. We then estimated the expected mortality from other infectious diseases as a result of ETEC-attributable or shigella-attributable stunting, hereafter referred to as indirect mortality. First, we used province-level data from the DHS^28^ to estimate the proportions of children with height-for-age Z scores less than 2 SDs (moderately stunted) or 3 SDs (severely stunted) below the mean height-for-age Z score. We constructed height-for-age Z score distributions *X*_s_ for each province using estimates of moderately and severely stunted children, then simulated a hypothetical height-for-age Z score distribution, *X*_s_ʹ, excluding the shift (∆) attributed to ETEC and shigella moderate-to-severe diarrhoeal episodes ((2.1), (2.2)).^17^

(2.1) $$X_{s}∼N(\mu ,\sigma^{2})$$

(2.2) $$X_{s}^{'}∼N(\mu ',\sigma^{2})\text{where}\mu '=\mu +\Delta$$

(2.3) $$Y_{a}=[(\mu -2\sigma )-(\mu -3\sigma )]-[(\mu '-2\sigma )-(\mu '-3\sigma )]$$

(2.4) $$Y_{b}=(\mu -3\sigma )-(\mu '-3\sigma )$$

We used the differences in probabilities of moderate stunting (*Y*_a_) and severe stunting (*Y*_b_) from the estimated and hypothetical distributions to calculate the increased fraction of moderately and severely stunted children in each province ((2.3), (2.4)).

We included country-specific child mortality rate estimates from 2016 WHO Maternal Child Epidemiology Estimation^24^ and GBD^25^ for diarrhoea, malaria, pneumonia, and measles—diseases for which childhood moderate and severe stunting is a known risk factor.^29^, ^30^ We used the population attributable risk (PAR) approach:

$$\text{PAR}=\frac{P_{e}({\text{RR}}_{e}-1)}{1+P_{e}({\text{RR}}_{e}-1)}$$

with fractions of ETEC-induced or shigella-induced moderate and severe stunting as the risk factor proportion *P*_e_ and the relative mortality risk RR_e_ associated with different moderate and severe stunting levels from Black et al.^30^

We estimated province-level mortality rates *d*_ID,s_ for each infectious disease, ID, and each province, *s,* as the product of national child mortality rates *D*_ID,c_ and the quotient from dividing DHS^28^ all-cause provincial mortality rates for children younger than 5 years, *U*_s_, by the mean national all-cause subnational mortality rate, *U*_c,_ from the DHS:

$$d_{\text{ID},s}=D_{\text{ID},c}.\frac{U_{s}}{U_{c}}$$

This ensured that the average of provincial infectious disease mortality rates within each country remained equal to national infectious disease mortality estimates. We report total mortality as the sum of direct mortality (from acute ETEC or shigella infection) and indirect mortality (other infectious disease deaths from ETEC-attributable or shigella-attributable stunting).

### Data and spatial analyses

We used complex survey design in Stata 14.2 for all statistical estimates, risk model imputations, and reported results, and report 95% CIs for our weighted means, calculated in Stata. We used ArcGIS version 10.6 (ESRI, Redlands, CA, USA) for all spatial analyses. We constructed the diarrhoeal mortality risk heat map by interpolating cluster-level weighted risk averages across 11 countries' surfaces using the empirical Bayesian kriging tool in the ArcGIS Spatial Analyst extension.^31^ We calculated the root squared mean error (RMSE) of several iterations of the empirical Bayesian kriging interpolation varying the input parameters, and included the one with the lowest RMSE here (appendix pp 2–4).^32^ The RMSE is the square root of the average difference between measured and predicted values and indicates how well predicted values correspond to measured values; ideally, the further away from 1 the RMSE, the better the model. The heat map is intended as a visualisation of risk over the study site.

### Sensitivity analysis

We did one-way sensitivity analyses in Sensit version 1.53 (TreePlan Software, San Francisco, CA, USA) to assess the impact of individually varying model inputs on our ETEC and shigella total mortality estimates (appendix p 7). Using SimVoi version 3.07 (TreePlan Software), we assessed the effect of simultaneous changes in multiple input variables through uncertainty analysis using Monte Carlo simulations. We ran our model for 10 000 iterations and estimated the upper (97·5%) and lower (2·5%) 95% uncertainty interveals (UIs) for key outputs (appendix p 11), which are reported along with our point estimates.

### Role of the funding source

The funder of the study had no role in study design, data collection, data analysis, data interpretation, or writing of the report. The corresponding author had full access to all of the data, and the final responsibility to submit for publication.

## Results

The percentage of moderate-to-severely underweight children younger than 5 years ranged from 11·2% to 35·7% by country (table 2); the Democratic Republic of the Congo (DRC), Ethiopia, and Burundi had the highest percentage. The percentage of children receiving preventive vitamin A was slightly higher (37·7–79·8%) than children receiving oral rehydration treatment (32·2–71·4%), although this pattern varied per country (table 2).

**Table 2:** Distribution of underlying diarrhoeal risk factors for children younger than 5 years across eastern and central African countries

		**Burundi**	**DRC**	**Ethiopia**	**Kenya**	**Malawi**	**Mozambique**	**Rwanda**	**Tanzania**	**Uganda**	**Zambia**	**Zimbabwe**
**Underweight**												
Number assessed		6063	8388	8975	18 909	5232	9830	3533	9052	4444	11 799	5012
Moderate to severe		35·7% (34·2–37·2)	26·3% (24·5–28·0)	28·4% (26·7–30·1)	14·8% (13·9–15·7)	15·1% (13·9–16·3)	18·4% (17·4–19·5)	12·0% (10·8–13·3)	18·0% (16·8–19·2)	12·8% (11·6–14·0)	19·1% (18·2–20·0)	11·2% (10·2–12·3)
Severe		8·1% (7·3–8·9)	6·3% (5·6–7·1)	6·1% (5·4–6·9)	2·3% (2·0–2·5)	1·7% (1·3–2·2)	3·6% (3·1–4·1)	2·0% (1·6–2·5)	2·4% (1·9–2·8)	2·0% (1·5–2·5)	3·0% (2·6–3·4)	1·4% (1·0–1·8)
**Access to care**												
Oral rehydration therapy												
	Number assessed	12 472	17 082	10 006	19 798	16 462	10 291	7364	9707	14 710	11 799	5807
	Received treatment	37·2% (36·8–37·5)	41·3% (40·6–42·0)	38·0% (37·3–38·6)	63·7% (63·5–63·9)	71·4% (71·3–71·6)	60·5% (59·7–61·3)	32·2% (32·0–32·4)	45·2% (44·6–45·8)	48·9% (48·5–49·3)	69·5% (69·3–69·8)	70·4% (69·9–70·9)
Vitamin A												
	Number assessed	12 439	16 947	9805	19 850	16 304	10 208	7352	9506	14 471	12 380	5731
	Received treatment	64·9% (63·2–66·5)	65·7% (63·6–67·9)	41·4% (39·0–43·8)	68·0% (66·8–69·2)	61·3% (60·0–62·6)	66·8% (65·0–68·6)	79·8% (78·7–80·9)	37·7% (35·9–39·6)	57·2% (55·7–58·7)	69·1% (67·7–70·5)	61·8% (59·3–64·2)
**Sanitation**												
Number assessed		13 192	18 715	10 641	20 958	17 286	11 102	7698	10 233	15 522	13 437	6132
Unimproved or no facility		58·8% (57·0–60·6)	82·5% (79·9–85·2)	90·0% (88·8–91·3)	78·8% (77·1–80·5)	49·4% (47·8–51·1)	80·5% (78·9–82·1)	42·1% (40·3–43·9)	85·1% (83·4–86·7)	95·6% (94·9–96·3)	77·2% (75·3–79·1)	68·2% (65·4–70·9)
Improved and unshared without a sewer connection		41·1% (39·3–42·9)	17·4% (14·7–20·0)	9·7% (8·4–10·9)	18·3% (16·8–19·8)	50·2% (48·6–51·8)	19·5% (17·9–21·1)	57·4% (55·6–59·2)	14·8% (13·2–16·4)	4·1% (3·4–4·7)	17·8% (16·2–19·4)	22·1% (19·6–24·6)
Improved and unshared with a sewer connection*		0·0% (0·0–0·1)	0·1% (0·0–0·3)	0·3% (0·1–0·4)	2·9% (2·0–3·7)	0·4% (0·0–0·8)	..	0·5% (0·3–0·7)	0·1% (0·0–0·2)	0·4% (0·2–0·5)	4·9% (3·8–6·1)	9·7% (8·0–11·5)
**Water source**												
Number assessed		13 192	18 709	10 641	20 964	17 286	11 102	7698	10 233	15 522	13 457	6132
Surface or unimproved		18·0% (15·9–20·2)	53·5% (49·0–58·1)	43·7% (38·8–48·5)	35·7% (33·6–37·7)	13·5% (11·8–15·3)	50·5% (47·1–53·9)	27·5% (24·7–30·2)	48·6% (45·3–52·0)	23·1% (20·7–25·5)	40·1% (37·7–42·6)	25·4% (22·0–28·8)
Off-plot improved		73·9% (71·5–76·2)	40·6% (36·3–45·0)	45·8% (41·2–50·3)	35·8% (33·7–38·0)	74·2% (72·1–76·2)	38·9% (35·5–42·3)	67·0% (64·2–69·8)	31·0% (28·1–33·9)	62·5% (60·0–65·1)	43·6% (40·7–46·4)	43·7% (40·3–47·0)
On-plot improved		8·1% (6·8–9·5)	5·8% (4·6–7·0)	10·6% (8·7–12·5)	28·5% (26·6–30·4)	12·3% (11·0–13·6)	10·6% (9·4–11·9)	5·5% (4·4–6·7)	20·4% (18·1–22·7)	14·3% (12·4–16·2)	16·3% (14·4–18·2)	30·9% (28·0–33·9)

Data are number of children assessed or weighted mean (95% CI) for each category. Weighted means and 95% CIs were calculated while accounting for survey design. DRC=Democratic Republic of the Congo.

* The Demographic and Health Survey for Mozambique did not include sewer connections when categorising sanitation facilities.

In all countries, low percentages of children lived in households with improved water and sanitation access, ranging from 0·0% (Mozambique) to 9·7% (Zimbabwe) for piped sanitation and from 5·5% (Rwanda) to 30·9% (Zimbabwe) for on-plot improved water (table 2). Less than 50% of children had access to improved, unshared sanitation in most countries, except for Rwanda and Malawi (table 2). Higher percentages of surveyed children had access to on-plot or off-plot improved water (46·4–86·5%) than improved sanitation (table 2).

**Figure 2:**
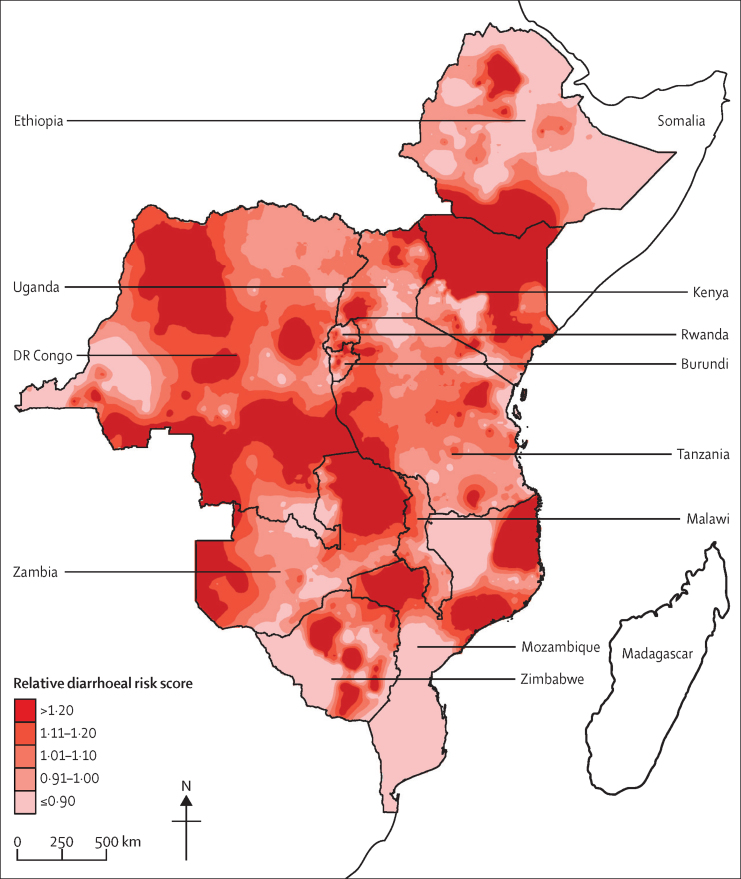
Heat map of relative diarrhoeal risk for children younger than 5 years across 11 eastern and central African countries Displayed surfaces are of spatial interpolations (predictions) estimated from cluster-level average diarrhoeal risk calculations using data from national Demographic and Health Surveys. Higher numbers indicate higher risk.

Overall diarrhoeal mortality risk was heterogeneous, with areas of higher and lower risk within each country (figure 2). Some countries had areas of marked concentrated risk (eg, Mozambique and Zimbabwe), whereas others had more diffuse patterns of risk over a large area (eg, Kenya and southern Ethiopia). The RMSE of this visualisation was 0·39.

**Figure 3:**
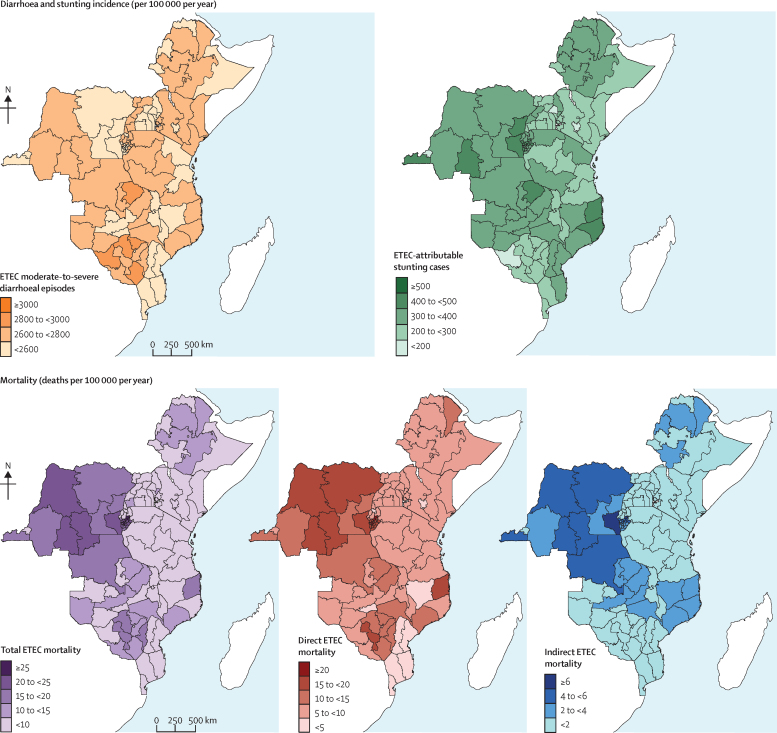
Maps of subnational estimates of ETEC burden, mortality, diarrhoeal episode incidence, and stunting incidence in children younger than 5 years across 11 eastern and central African countries Mortality and incidence are given per 100 000 children younger than 5 years per year. Total mortality burden is the sum of direct and indirect mortality rates, where indirect mortality is deaths from other infectious diseases from ETEC-attributable stunting. ETEC=enterotoxigenic *Escherichia coli*.

**Figure 4:**
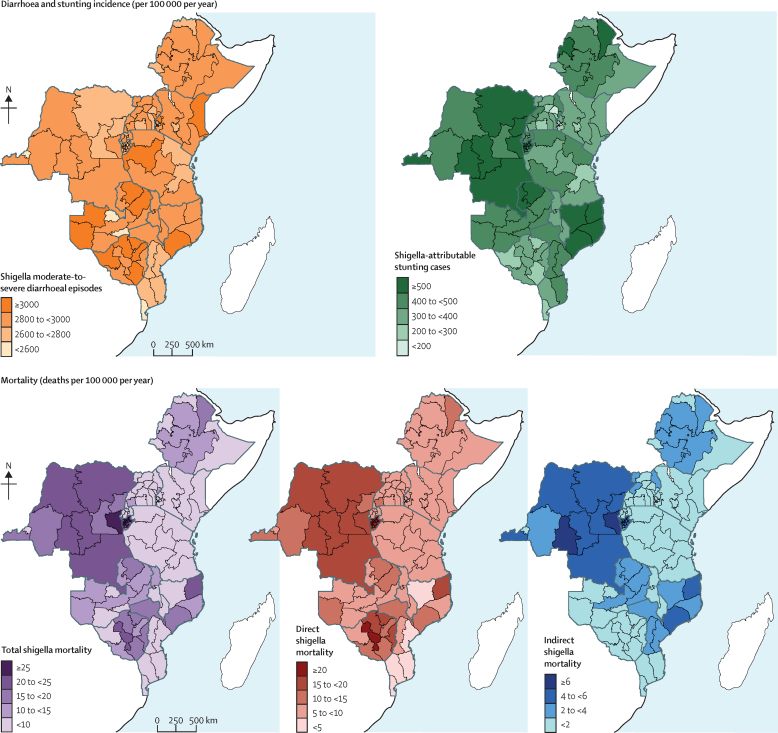
Maps of subnational estimates of shigella burden, mortality, diarrhoeal episode incidence, and stunting incidence in children younger than 5 years across 11 eastern and central African countries Mortality and incidence are given per 100 000 children younger than 5 years per year. Total mortality burden is the sum of direct and indirect mortality rates, where indirect mortality is deaths from other infectious diseases from shigella-attributable stunting. Shigella estimates had a higher range of values than ETEC estimates because of its higher aetiological fraction and Z score shift. ETEC=enterotoxigenic *Escherichia coli*.

Children with the highest annual incidence of ETEC moderate-to-severe diarrhoeal episodes were from four Zimbabwean provinces—Mashonaland Central and West, Matabeleland North, and Masvingo, ranging from 2804 episodes (95% UI 923–4321) to 2879 episodes (947–4436) per 100 000 children—and from the Northern province in Zambia, with 2803 episodes (923–4320) per 100 000 children (figure 3; appendix pp 12–20). Children with the highest annual incidence of shigella moderate-to-severe diarrhoeal episodes were found in the provinces mentioned above, as well as Mashonaland East in Zimbabwe (3008 episodes [1012–4535]); Muchinga, North Western, and Western provinces in Zambia (ranging from 3015 episodes [1014–4544] to 3065 episodes [1031–4619]); Western province in Tanzania (3023 episodes [1017–4556]); Zambezia province in Mozambique (3010 episodes [1013–4538]); and North Eastern (3040 episodes [1023–4583]) and Western (3005 episodes [1011–4530]) provinces in Kenya (figure 4; appendix pp 21–28).

Although there was some subnational heterogeneity in moderate-to-severe diarrhoeal episodes for either disease within most countries (Figure 3, Figure 4), it was small. For countries other than Zimbabwe, the provinces with the highest episode incidences were usually less than 1·5 times the incidence of the province with the lowest incidence (appendix pp 12–28). In Zimbabwe, the episode incidence was almost 1·8 times greater in Mashonaland Central compared with Bulawayo (appendix pp 12–28).

Children with the highest annual incidence of ETEC-attributable stunting cases were from half of Burundi's provinces distributed throughout the north (ranging from 403 cases [95% UI 131–631] to 460 [150–721] cases per 100 000 children), four of DRC's provinces (Bas Congo, Kasai Occidental, Nord Kivu, and Sud Kivu, ranging from 415 cases [136–650] to 456 cases [149–715] per 100 000 children), and two northeastern provinces from Mozambique (421 cases [138–660] per 100 000 children in Cabo Delago and 420 [137–658] in Nampula; figure 3; appendix pp 12–20). For shigella, children with highest stunting incidence rates were from the same provinces as those with the highest ETEC stunting rates, followed closely by Afar and Benishangul-Gumuz (507 cases [167–775] per 100 000 children for both) in Ethiopia, Niassa (524 cases [172–800]), and Zambezia (517 cases [170–790]) in Mozambique, and Luapula (507 cases [167–775]) and Northern province (526 cases [173–803]) in Zambia (figure 4; appendix pp 21–28).

Most study countries showed subnational variation in ETEC-attributable and shigella-attributable stunting incidence, with Mozambique, DRC, and Uganda having the greatest variation (Figure 3, Figure 4). In Mozambique, children in the northwestern two provinces had the highest stunting incidence rates for either disease, and this decreased going southwest, with the southernmost province having the lowest stunting incidence (Figure 3, Figure 4). Within DRC, most subnational areas had rates equivalent to the national estimates, except for Kinshasa's stunting incidence being less than half of the national stunting incidence. Within Uganda, provinces on the eastern and western borders had higher rates than the central provinces (Figure 3, Figure 4).

The highest annual direct ETEC mortality rates for children were in 16 of 18 Burundi provinces (ranging from 15·2 deaths [10·4–20·7] to 23·9 deaths [16·4–32·6] per 100 000 children); followed by five provinces in north to central DRC (15·5 deaths [10·6–21·1] to 16·9 deaths [11·6– 23·0]); a central province (Midlands) in Zimbabwe (18·6 deaths [12·7–25·3]); and the northeastern province of Cabo Delgado in Mozambique (15·8 deaths [10·9–21·6]; figure 3; appendix pp 12–20). Children in these provinces also had high direct shigella mortality, along with children from two additional provinces in central and south DRC and two additional central provinces in Zimbabwe (≥15 deaths per 100 000 children; figure 4; appendix pp 21–28). Overall, direct mortality ranged from 1·5 (UI 1·0–2·0) to 23·9 (16·4–32·6) deaths per 100 000 children for ETEC and 1·6 (1·2–2·1) to 26·2 (19·0–34·4) deaths per 100 000 children for shigella.

The provinces with the highest annual indirect child mortality rates were Kirundo (ETEC: 6·2 deaths [UI 2·0–9·6] and shigella: 8·1 deaths [2·7– 12·3] per 100 000 children) in Burundi; and Sud Kivu (ETEC: 6·0 deaths [2·0– 9·3] and shigella: 7·9 deaths [2·6– 11·9]) and Kasai Occidental (ETEC: 5·8 deaths [1·9– 8·9] and shigella: 7·5 deaths [2·5–11·4]) in DRC. These were followed closely by several other provinces in Burundi and DRC (Figure 3, Figure 4; appendix pp 12–28).

On average across the 110 provinces, indirect mortality accounted for 15·7% (ETEC) and 18·2% (shigella) of the total burden. For many provinces in Burundi, DRC, Ethiopia, and Mozambique, indirect mortality accounted for a quarter to a third of the total mortality (appendix pp 12–28).

ETEC and shigella total mortality annual rates were highest for children in Burundi (>20 deaths per 100 000 children in 14 provinces for ETEC and 16 provinces for shigella; Figure 3, Figure 4). For ETEC total mortality, other provinces with high annual mortality rates for children were almost half of the 11 provinces in DRC, Cabo Delgado in Mozambique, and Midlands in Zimbabwe (figure 3; appendix pp 12–20). These areas also had high shigella total mortality rates for children, with the addition of two provinces in DRC (figure 4; appendix pp 21–28).

Mozambique, DRC, and Zimbabwe displayed high subnational variation in most ETEC and shigella mortality measures, while Kenya, Tanzania, Ethiopia, and Uganda had no to little variation in mortality measures (Figure 3, Figure 4). Mozambique had the highest heterogeneity in ETEC and shigella direct mortality rates, with the central estimates of three provinces (Cabo Delgado, Tete, and Zambezia) having rates at least 1·5 times the national rate estimates. Cabo Delgado, the province with the highest rates in Mozambique, had a central rate estimate almost five times higher than its neighbouring province with the lowest rate estimate, Niassa (appendix pp 12–28). Mozambique also had the highest heterogeneity in indirect death rate estimates. Children in Cabo Delgado and Zambezia provinces had central indirect death rate estimates 1·6–1·8 higher than national rates and 4–5 times higher, respectively, than Maputo, the province with the lowest central rate estimate (appendix pp 12–28). Within DRC, variation existed in direct ETEC and shigella mortality rates, displaying a similar pattern to the stunting pattern described above (Figure 3, Figure 4). However, children in DRC from Sud Kivu and Kasai Occidental had central indirect mortality rate estimates from disease-attributable stunting 1·5 times the national rate estimate (appendix pp 12–28). In Zimbabwe, there was heterogeneity in direct ETEC and shigella mortality rates but not in indirect mortality rates. Children from Midlands had a central ETEC mortality rate estimate 1·5 times higher than the national rate estimate, and more than five times higher than the province with the lowest central rate estimate in Zimbabwe, Bulawayo (appendix pp 12–28).

For most countries, children in the two lowest wealth quintiles were more likely to have higher risk index values than children from the highest wealth quintile, both in rural and urban settings (appendix p 29). Rural households had higher risk indices than the urban households for their corresponding wealth quintile in all countries (appendix p 29). There was a larger disparity in risk between children from the highest and lowest wealth quintiles in urban areas compared with their counterparts in rural areas. On average, children from urban households in the lowest quintile had three times the risk of children in the highest quintile, with some countries having a notably higher disparity (eg, Burundi and Ethiopia). On average, the children from rural households in the lowest quintile had twice the risk as compared with those in rural households in the highest quintile, and one country (Mozambique) had a higher disparity (appendix p 29).

The variables with the greatest effect on the total ETEC and shigella mortality burden across the 110 provinces in this study were the molecular adjustments (swing of 56·2% [ETEC] and 41·2% [shigella] from base case estimates); the aetiological fractions attributed to each pathogen (32·3% [ETEC] and 41·2% [shigella] from base case estimates); and the fraction of moderate-to-severe diarrhoeal episodes that cause a height-for-age Z score shift (6·1% [ETEC] and 10·5% [shigella] from base case estimates; appendix p 8). The variables with the greatest effect on cases of moderate and severe stunting were the fraction of moderate-to-severe diarrhoeal episodes that cause a height-for-age Z score shift (61·0% [ETEC] and 65·9% [shigella] from base case estimates); molecular adjustments (17·0% [ETEC] and 10·5% [shigella] from base case estimates); and the aetiological fractions attributed to each pathogen (9·8% [ETEC] and 10·5% [shigella] from base case estimates).

## Discussion

To the best of our knowledge, this is the first study to quantify ETEC and shigella morbidity and total mortality, both from infection and from the consequences of diarrhoea-associated stunting, at the subnational level for several sub-Saharan African countries. This is a scale of analysis little explored by current diarrhoeal research but crucial to improving intervention strategies. Quantifying heterogeneity identified high-burden provinces with relatively low ETEC and shigella mortality from acute infection, but with total ETEC and shigella mortality estimates a quarter to a third higher once infectious disease deaths from ETEC-attributable and shigella-attributable stunting were included.

Our metrics identified that the highest total mortality rates for ETEC and shigella were in Burundi; most provinces of Burundi had the highest incidences of stunting across our study countries. Many DRC provinces also had high total ETEC and shigella mortality rates and high incidences of stunting. High morbidity and mortality rates from ETEC and shigella infections in these countries were probably driven by the high percentage of moderately-to-severely underweight children (eg, 35·7% of children younger than 5 years in Burundi and 26·3% in DRC).

Our subnational analysis, which included indirect burden measures, identified geographical areas with high mortality from ETEC-attributable and shigella-attributable stunting that would have been less apparent if our models had only included direct diarrhoeal mortality. We also identified areas of high direct mortality burden within Zimbabwe and other countries that would have been undetected at a national scale. For example, when assessed at a national level, children from Zimbabwe had lower risk compared with children from the other study countries. However, our analysis identified Midlands as having higher direct burden than almost all the other provinces. A recent study using a high-resolution Bayesian geostatistical model found this area in Zimbabwe to be one of only five subnational areas within Africa with an increase in diarrhoeal mortality from 2000 to 2015.^11^ These observations taken together confirm the importance of assessing the total mortality envelope at different spatial scales. Understanding within-country heterogeneity can help to distinguish subpopulations that will benefit most from water, sanitation, and hygiene (WASH) interventions and deployment of enteric vaccines, while also identifying communities to study further on cause-specific risk and burden. Our findings corroborate others^11^ that even within high-risk and high-burden countries of the world, certain subpopulations bear a disproportionate amount of burden.

By including indirect burden, our total ETEC and shigella mortality estimates increased by an average of 15·7% and 18·0%, respectively—a conservative increase compared with other global studies^6^, ^7^ that included indirect burden associated with other diarrhoeal causes in their estimates at broader spatial scales. For the provinces in our study, Reiner and colleagues^11^ reported overall direct diarrhoeal mortality rates that ranged from 100 to 150 deaths per 100 000 children. Our estimates (after applying our assumed aetiological fractions) fit within the range of these model outputs with direct rates of 1·5 (UI 1·0–2·0) to 23·9 (16·4–32·6) deaths per 100 000 children for ETEC and 1·6 (1·2–2·1) to 26·2 (19·0–34·4) deaths per 100 000 children for shigella.

The overall pattern of subnational heterogeneity in mortality rates in our study and Reiner and colleagues^11^ was similar for all study countries, except for Mozambique. Although we used the same DHS as our basis of analysis, the main reason for this discrepancy is probably because of the methods employed: we used child-oriented and exposure-related variables to distribute our estimates, and these regions of Mozambique have high exposure risk and high under-5 mortality. We offer our estimates as a different way of viewing diarrhoeal burden. Future subnational studies should explore diarrhoeal mortality and morbidity burden by cause across larger high-risk regions to capture local variation in aetiological-specific burden, as pathogens with different ecologies could thrive in different environmental conditions. Additionally, future assessment of subnational total burden related to ETEC and shigella infection should incorporate other ecological and environmental covariates to produce a more accurate high-resolution spatial projection.

Osgood-Zimmerman and colleagues^33^ assessed spatiotemporal trends in child growth failure at high spatial resolution across sub-Saharan Africa using prevalence data from the DHS and other sources. Although many of our provinces with high rates of ETEC and shigella stunting had a high prevalence of moderate and severe stunting in their study (eg, Nord Kivu, Sud Kivu, and Maniema in DRC and many provinces in Burundi), observed differences between our results and theirs in Ethiopia, Zambia, and Kenya can potentially be attributed to the dates of the surveys used (2010 Ethiopia survey *vs* 2016 in our study) and different organisation of subnational demarcations (Zambia and Kenya). Indeed, as Osgood-Zimmerman and colleagues discussed,^33^ Ethiopia showed substantial decreases in child undernutrition from 2005 to 2015.^15^ The largest dissimilarity in our results are for certain regions in Mozambique (eg, Cabo Delgado and Nampula for ETEC and all four northeastern Mozambique provinces for shigella). Our results show high prevalence of ETEC and shigella stunting, which Osgood-Zimmerman and colleagues^33^ have shown to be in the mid-range of stunting for these regions. This difference is due to our use of child exposure risk as explained above, which results in a higher likelihood of experiencing diarrhoea and its downstream effects according to our model.

All our shigella burden estimates were higher than our ETEC estimates because of its higher aetiological fraction than ETEC; this difference was even more pronounced in our stunting and indirect mortality estimates because, in addition to the higher aetiological fraction, there also is a higher Z score shift associated with shigella episodes. In Khalil and colleagues,^19^ they observed a similar pattern, where their shigella national mortality estimates were higher than their ETEC national mortality estimates for the same reason.

While there was substantial variation in risk, our supplemental analysis of risk index by economic status and household setting (appendix p 29) indicated that these are additional influential factors that should be considered when assessing risk and burden heterogeneity. Most notably, household economic status is a clear indirect factor in determining risk. Its effects are the results of combined inequality in underlying undernutrition, health-care access, and ecological exposure variables. Future investigators should also evaluate the impact and cost-effectiveness of ETEC and shigella vaccination interventions subnationally. These differences are important to consider when planning effective interventions and are likely to affect the impact and cost-effectiveness of vaccination.

Although our model relies on well characterised and established factors related to susceptibility and exposure, we used proxy measures for risk and do not have aetiological confirmation of diarrhoeal episodes for DHS-surveyed children. Additionally, we used data on oral rehydration treatment use (oral rehydration solution and recommended home solution) in assessing differences across subpopulations, yet we relied on oral rehydration solution effectiveness for determining the relative effect of those differences in behaviours. This is because, to our knowledge, there is no robust estimate of the effectiveness of recommended home solution compared with oral rehydration solution. Because recommended home solution is more common in more marginalised subpopulations, leaving it out of the analysis would overstate the disparity between marginalised and more privileged subpopulations. In addition, there are other differences in treatment (eg, antibiotic availability, treatment timing, zinc supplementation) that we did not account for in our model, because full data are not available on the use and effects of these risk factors. Current methods of attributing aetiological causes of diarrhoea are not perfect; while traditional culture-based methods underestimate the presence of pathogens, molecular methods might overestimate them. As we used molecularly adjusted aetiological fractions in our study, we might be overestimating their presence. Although there is growing availability of country-level modelled estimates of diarrhoeal episode incidence, we relied on WHO regional-level estimates for this analysis. We did so to increase transparency and avoid adding additional model uncertainty to our estimates, but this probably understates episode heterogeneity across countries. Also, the similarities between the distribution of ETEC and shigella burden are a result of our assumption that factors contributing to increased mortality were equivalent for ETEC and shigella, in light of the lack of data to differentiate between the mortality risk attributed to infection by these pathogens. Improved estimates of the differential burden including case fatality rates of the two pathogens could greatly improve public health decision making about vaccines or WASH in these high-risk and resource-constrained settings. Our subnational estimates would be improved by longitudinal studies of community-level infection patterns of ETEC and shigella, which could help to validate our model's assumptions and refine burden estimates. While the DHS's cluster-based sampling survey design provides an unevenly distributed dataset not ideal for basic spatial interpolations, we implemented a previously used^32^ interpolation approach that can be used to effectively project DHS data. Our heat map of risk is a visualisation tool, and its RMSE is higher than other diarrhoeal maps.^11^ In future studies, inclusion of spatial covariates in our interpolation models might improve their fit. Anderson and colleagues^8^ provided specific caveats for our methods of burden estimation.

We showed that assessing diarrhoeal disease risk and ETEC and shigella infection-associated morbidity and total mortality at a subnational level can identify previously undetected high-burden areas. Our emphasis on provincial differences identified potentially susceptible populations that would benefit most from interventions and that would not have been identified through traditional measures, providing country-level decision makers with valuable information to help to prioritise the implementation of interventions in resource-constrained settings.

## References

[1] Liu L, Oza S, Hogan D (2016). Global, regional, and national causes of under-5 mortality in 2000–15: an updated systematic analysis with implications for the Sustainable Development Goals. Lancet.

[2] Fischer Walker CL, Perin J, Martin J, Bochi-Pinto C, Black RE (2012). Diarrhea incidence in low- and middle-income countries in 1990 and 2010: a systematic review. BMC Public Health.

[3] Guerrant RL, DeBoer MD, Moore SR, Scharf RJ, Lima AAM (2012). The impoverished gut—a triple burden of diarrhoea, stunting and chronic disease. Nat Rev Gastroenterol Hepatol.

[4] Checkley W, Buckley G, Gilman RH (2008). Multi-country analysis of the effects of diarrhoea on childhood stunting. Int J Epidemiol.

[5] Black RE, Victora CG, Walker SP (2013). Maternal and child undernutrition and overweight in low-income and middle-income countries. Lancet.

[6] Troeger C, Colombara DV, Rao PC (2018). Global disability-adjusted life-year estimates of long-term health burden and undernutrition attributable to diarrhoeal diseases in children younger than 5 years. Lancet Glob Health.

[7] Khalil IA, Troeger C, Rao PC (2018). Morbidity, mortality, and long-term consequences associated with diarrhoea from *Cryptosporidium* infection in children younger than 5 years: a meta-analyses study. Lancet Glob Health.

[8] Anderson JD, Bagamian KH, Muhib F (2019). Burden of enterotoxigenic *Escherichia coli* and shigella non-fatal diarrhoeal infections in 79 low-income and lower middle-income countries: a modelling analysis. Lancet Glob Health.

[9] Wang H, Bhutta ZA, Coates MM (2016). Global, regional, national, and selected subnational levels of stillbirths, neonatal, infant, and under-5 mortality, 1980–2015: a systematic analysis for the Global Burden of Disease Study 2015. Lancet.

[10] Penny MA, Verity R, Bever CA (2016). Public health impact and cost-effectiveness of the RTS, S/AS01 malaria vaccine: a systematic comparison of predictions from four mathematical models. Lancet.

[11] Reiner RC, Graetz N, Casey DC (2018). Variation in childhood diarrheal morbidity and mortality in Africa, 2000–2015. N Engl J Med.

[12] Hay SI, Okiro EA, Gething PW (2010). Estimating the global clinical burden of *Plasmodium falciparum* malaria in 2007. PLoS Med.

[13] Joshi AB, Luman ET, Nandy R, Subedi BK, Liyanage JB, Wierzba TF (2009). Measles deaths in Nepal: estimating the national case-fatality ratio. Bull World Health Organ.

[14] Azage M, Kumie A, Worku A, Bagtzoglou AC (2015). Childhood diarrhea exhibits spatiotemporal variation in northwest Ethiopia: a SaTScan spatial statistical analysis. PLoS One.

[15] Troeger C, Forouzanfar M, Rao PC (2017). Estimates of global, regional, and national morbidity, mortality, and aetiologies of diarrhoeal diseases: a systematic analysis for the Global Burden of Disease Study 2015. Lancet Infect Dis.

[16] Liu J, Platts-Mills JA, Juma J (2016). Use of quantitative molecular diagnostic methods to identify causes of diarrhoea in children: a reanalysis of the GEMS case-control study. Lancet.

[17] Kotloff KL, Nataro JP, Blackwelder WC (2013). Burden and aetiology of diarrhoeal disease in infants and young children in developing countries (the Global Enteric Multicenter Study, GEMS): a prospective, case-control study. Lancet.

[18] Rogawski ET, Liu J, Platts-Mills JA (2018). Use of quantitative molecular diagnostic methods to investigate the effect of enteropathogen infections on linear growth in children in low-resource settings: longitudinal analysis of results from the MAL-ED cohort study. Lancet Glob Health.

[19] Khalil IA, Troeger C, Blacker BF (2018). Morbidity and mortality due to shigella and enterotoxigenic *Escherichia coli* diarrhoea: the Global Burden of Disease Study 1990–2016. Lancet Infect Dis.

[20] World Bank (2017). Reducing inequalities in water supply, sanitation, and hygiene in the era of the Sustainable Development Goals: synthesis report of the WASH poverty diagnostic initiative.

[21] USAID The DHS program: demographic and health surveys. https://dhsprogram.com/data.

[22] Vos T, Abajobir AA, Abate KH (2017). Global, regional, and national incidence, prevalence, and years lived with disability for 328 diseases and injuries for 195 countries, 1990–2016: a systematic analysis for the Global Burden of Disease Study 2016. Lancet.

[23] Prüss-Ustün A, Bartram J, Clasen T (2014). Burden of disease from inadequate water, sanitation and hygiene in low- and middle-income settings: a retrospective analysis of data from 145 countries. Trop Med Int Health.

[24] Maternal and Child Health Estimation group WHO-MCEE estimates for child causes of death, 2000–2015. http://www.who.int/healthinfo/global_burden_disease/en.

[25] Institute for Health Metrics and Evaluation (2016). GBD Compare. Viz Hub. http://vizhub.healthdata.org/gbd-compare.

[26] Lanata CF, Fischer-Walker CL, Olascoaga AC (2013). Global causes of diarrheal disease mortality in children <5 years of age: a systematic review. PLoS One.

[27] Fischer Walker CL, Rudan I, Liu L (2013). Global burden of childhood pneumonia and diarrhoea. Lancet.

[28] USAID Demographic and Health Survey (DHS) STATcompiler. http://www.statcompiler.com/en.

[29] Caulfield LE, de Onis M, Blössner M, Black RE (2004). Undernutrition as an underlying cause of child deaths associated with diarrhea, pneumonia, malaria, and measles. Am J Clin Nutr.

[30] Black RE, Allen LH, Bhutta ZA (2008). Maternal and child undernutrition: global and regional exposures and health consequences. Lancet.

[31] Pilz J, Spöck G (2008). Why do we need and how should we implement Bayesian kriging methods. Stoch Environ Res Risk Assess.

[32] Jia P, Anderson JD, Leitner M, Rheingans R (2016). High-resolution spatial distribution and estimation of access to improved sanitation in Kenya. PLoS One.

[33] Osgood-Zimmerman A, Millear AI, Stubbs RW (2018). Mapping child growth failure in Africa between 2000 and 2015. Nature.

