# Supplementary material for: Heterogeneity in enterotoxigenic Escherichia coli and shigella infections in children under 5 years of age from 11 African countries: a subnational approach quantifying risk, mortality, morbidity, and stunting
Source: Lancet Glob Health. 2019 Nov 14;8(1):e101–12. doi: 10.1016/S2214-109X(19)30456-5 (PMC7024994; doi:10.1016/S2214-109X(19)30456-5)
Supplement: Supplementary appendix [file mmc1.pdf]

THE LANCET
Global Health

Supplementary appendix

This appendix formed part of the original submission and has been peer reviewed. We post it as supplied by the authors.

Supplement to: Bagamian KH, Anderson JD, Muhib F, et al. Heterogeneity in enterotoxigenic *Escherichia coli* and shigella infections in children under 5 years of age from 11 African countries: a subnational approach quantifying risk, mortality, morbidity, and stunting. *Lancet Glob Health* 2019; published online Nov 13. http://dx.doi.org/10.1016/S2214-109X(19)30456-5.

Contents

Supplemental Methods 2

Exposure 2

Susceptibility 2

Spatial Interpolation 3

Supplementary Figure 1. Maps of countries from East and Central Africa included in study. 5

Supplemental Figure 2. Analysis work flow diagram 7

Supplemental Figure 3. Tornado plots of key input variables on ETEC and Shigella morbidity and mortality estimates. 8

Supplementary Table 1. Relative risk values from literature and Demographic and Health survey (DHS) data used to calculate exposure (A) and susceptibility (B) indices. 9

Supplementary Table 2. Exposure scenarios and assigned relative risks from literature estimates. 10

Supplementary Table 3. Key assumptions and inputs in calculating ETEC and *Shigella* burden. 11

Supplementary Table 4. National and subnational ETEC morbidity and mortality estimates for under-five children in 2016 for 11 eastern and central African countries. 12

Supplementary Table 5. National and subnational Shigella morbidity and mortality estimates for under-five children in 2016 for 11 eastern and central African countries. 21

Supplementary Table 6. Distribution of risk indices for under-five children across eastern and central African countries by economic status and household setting (urban or rural). 29

Supplemental Methods

Exposure

We determined the exposure relative risks (Table ST1) using a systematic review-based meta-analysis of various water and sanitation hygiene (WASH) interventions corresponding to the different exposure scenarios[1] (Table ST2). Our chosen exposure scenarios for our analyses have RR values based on rigorous studies. We excluded 'point of use' water treatment scenarios because of the challenge of estimating adequate compliance and the questionable reliability of the RR estimates.[2] Previous efforts have assigned RRs to various WASH exposure scenarios, but have only distinguished between one or two levels of water and sanitation service.[3,4] Here, we considered multiple service levels or 'exposure' scenarios, as this approach can distinguish between, for example, improved sanitation with and without a sewer connection, allowing for different RRs of a given health outcome for each exposure level. We used the exposure scenarios and associated diarrheal disease RRs for access to drinking water and sanitation proposed by Wolf and colleagues,[1] (Table ST2).

Susceptibility

We estimated undernutrition RRs based on z-scores assigned to under-five children in the child-level DHS data (Table ST2). RRs are assigned to different levels of weight-for-age (WFA) based on standard deviations (SD) below the mean of the z-score distribution (-1 to -2 SD [mild], -2 to -3 SD [moderate], and less than -3 SD [severe]) compared to normal (greater than -1 SD).[5] We use published RRs for each level[5] to estimate a piece-wise linear risk function that provides a continuous estimate of excess risk as WFA z-scores decline (Table ST2). We include oral rehydration therapy (ORT) in our model as there is substantial evidence of the effect of oral rehydration solution (ORS) on the severity and duration of diarrhea; a recent systematic review of 157 studies estimated a 93% reduction in diarrhea mortality with ORS use.[6] We combined this estimate with an estimated probability of receiving ORT (including both ORS and home-remedy rehydration solution [RHS]), which was calculated using child-level DHS data

(Table ST2). To the best of our knowledge, there is not a comparable estimate of mortality reduction with RHS use as there is for ORS use, but both therapies are recommended by the WHO and UNICEF.[7]

We included vitamin A in our model as a meta-analysis of 12 studies of diarrhea specific mortality estimated a pooled effect of ~30% mortality reduction due to vitamin A supplementation (RR=0.70; CI: 0.58–0.86) among children 6-59 months of age.[8] This estimate is incorporated by using child-level DHS data on whether the child received a vitamin A dose.

Spatial Interpolation

Of 7,699 household clusters, we excluded those without available geographic coordinates (n=90) or with fewer than five children sampled (n=2,486) from the spatial interpolation dataset. We adjusted risk by dividing each individual child risk score by the average risk of children from their survey cluster. The average adjusted individual child risk per cluster were the interpolation input values. We used the empirical Bayesian kriging (EBK) tool in the ArcGIS Spatial Analyst extension[9] to implement the interpolation. We used this method because it uses the underlying data distribution to build a continuous surface predicted from the input data values (a better approach to calculating estimates for an irregularly clustered dataset[9] such as the DHS). When applied to DHS data, EBK-based interpolations produce a minimal root mean square error (RMSE) as compared with other interpolation methods.[10] The RMSE is the square root of the average difference between measured and predicted values and indicates how well predicted values correspond to measured values; ideally, the further away from 1 the RMSE, the better the model. We calculated the RMSE of several iterations of the interpolation with different input parameters and included the one with the lowest RMSE here. To protect survey respondents’ identities, the DHS randomly displaces geographic coordinates by up to 2 km for urban clusters and by up to 5 km for rural clusters (with 1% of rural clusters being displaced by 10 km).[11] The spatial resolution of our output was at 15 km—larger than the potential cluster displacement distance.

References

1 Wolf J, Prüss-Ustün A, Cumming O, *et al.* Systematic review: Assessing the impact of drinking water and sanitation on diarrhoeal disease in low- and middle-income settings: systematic review and meta-regression. *Tropical Medicine & International Health* 2014; **19**: 928–42.

2 Schmidt W-P, Cairncross S. Household Water Treatment in Poor Populations: Is There Enough Evidence for Scaling up Now? *Environ Sci Technol* 2009; **43**: 986–92.

3 Prüss A, Kay D, Fewtrell L, Bartram J. Estimating the burden of disease from water, sanitation, and hygiene at a global level. *Environ Health Perspect* 2002; **110**: 537–42.

4 Cairncross S, Valdmanis V. Water Supply, Sanitation, and Hygiene Promotion. In: Jamison DT, Breman JG, Measham AR, *et al.*, eds. Disease Control Priorities in Developing Countries, 2nd edn. Washington (DC): World Bank, 2006: 771–92.

5 Caulfield LE, de Onis M, Blössner M, Black RE. Undernutrition as an underlying cause of child deaths associated with diarrhea, pneumonia, malaria, and measles. *Am J Clin Nutr* 2004; **80**: 193–8.

6 Munos MK, Walker CLF, Black RE. The effect of oral rehydration solution and recommended home fluids on diarrhoea mortality. *Int J Epidemiol* 2010; **39 Suppl 1**: i75-87.

7 Organization WH. Clinical management of acute diarrhoea: WHO. Geneva: World Health Organization, 2004.

8 Imdad A, Yakoob MY, Sudfeld C, Haider BA, Black RE, Bhutta ZA. Impact of vitamin A supplementation on infant and childhood mortality. *BMC Public Health* 2011; **11 Suppl 3**: S20.

9 Pilz J, Spöck G. Why do we need and how should we implement Bayesian kriging methods. *Stochastic Environmental Research and Risk Assessment* 2008; **22**: 621–32.

10 Jia P, Anderson JD, Leitner M, Rheingans R. High-Resolution Spatial Distribution and Estimation of Access to Improved Sanitation in Kenya. *PloS one* 2016; **11**: e0158490.

11 USAID. The DHS Program: Demographic and Health Surveys. https://dhsprogram.com/data/.

Supplementary Figure 1. Maps of countries from East and Central Africa included in study.

A. Location of clusters (n=7590) from DHS surveys (grey dots). B. Subnational divisions of study countries.

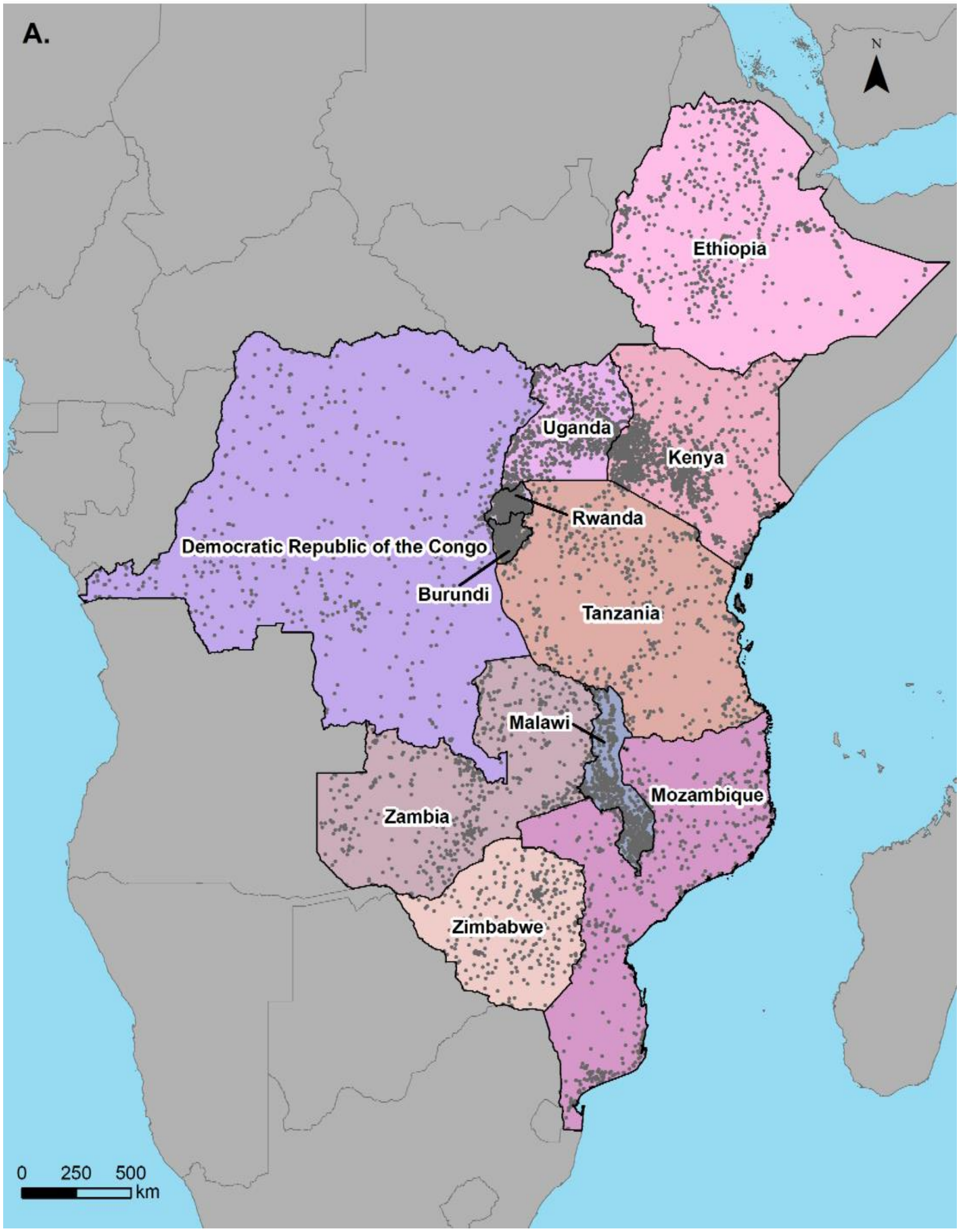

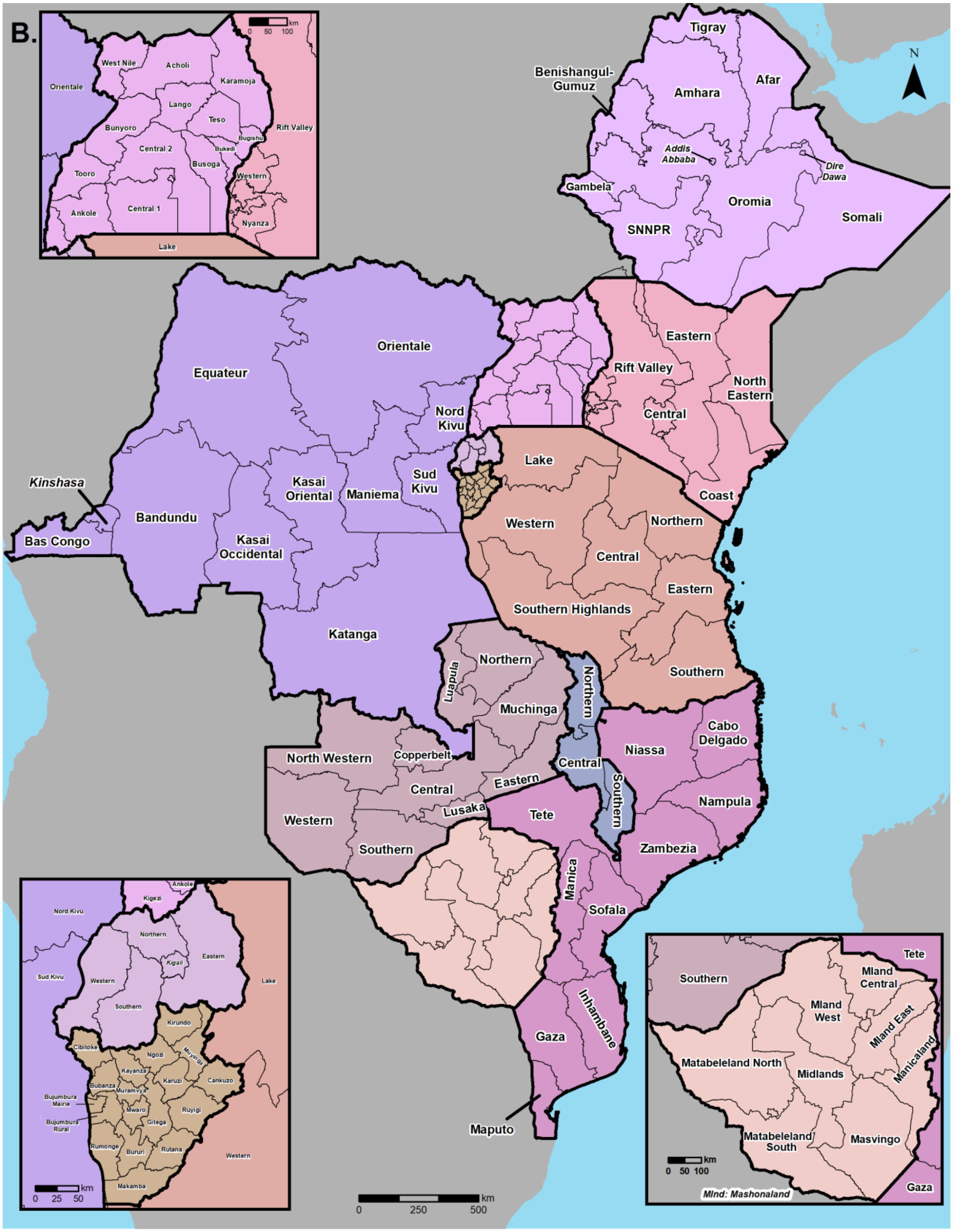
B.
N
Tigray
Afar
Amhara
Benishangul-Gumuz
Addis Abbaba
Dire Dawa
Gambela
Oromia
Somali
SNNPR
Orientale
Equateur
Nord Kivu
Sud Kivu
Kinshasa
Bas Congo
Bandundu
Kasai Oriental
Maniema
Kasai Occidental
Katanga
Eastern
Rift Valley
North Eastern
Central
Coast
Lake
Western
Northern
Central
Eastern
Southern Highlands
Southern
Luapula
Northern
Muchinga
Northern
Central
Southern
North Western
Copperbelt
Central
Eastern
Lusaka
Western
Southern
Cabo Delgado
Niassa
Nampula
Tete
Zambezia
Manica
Sofala
Inhambane
Gaza
Maputo
West Nile
Acholi
Karamoja
Orientale
Lango
Teso
Rift Valley
Bunyoro
Central 2
Bugishu
Bukedi
Busoga
Tooro
Western
Ankole
Central 1
Nyanza
Lake
km
0 50 100
Ankole
Kigezi
Nord Kivu
Northern
Eastern
Kigali
Sud Kivu
Western
Lake
Southern
Kirundo
Cibitoke
Ngozi
Muyinga
Kayanza
Bubanza
Muramvya
Karuzi
Cankuzo
Bujumbura Mairie
Mwaro
Ruyigi
Bujumbura Rural
Gitega
Rumonge
Bururi
Rutana
Western
Makamba
km
0 25 50
km
0 250 500
Tete
Southern
Mland Central
Mland West
Mland East
Manicaland
Matabeleland North
Midlands
Matabeleland South
Masvingo
Gaza
km
0 50 100
Mlnd: Mashonaland

Supplemental Figure 2. Analysis work flow diagram

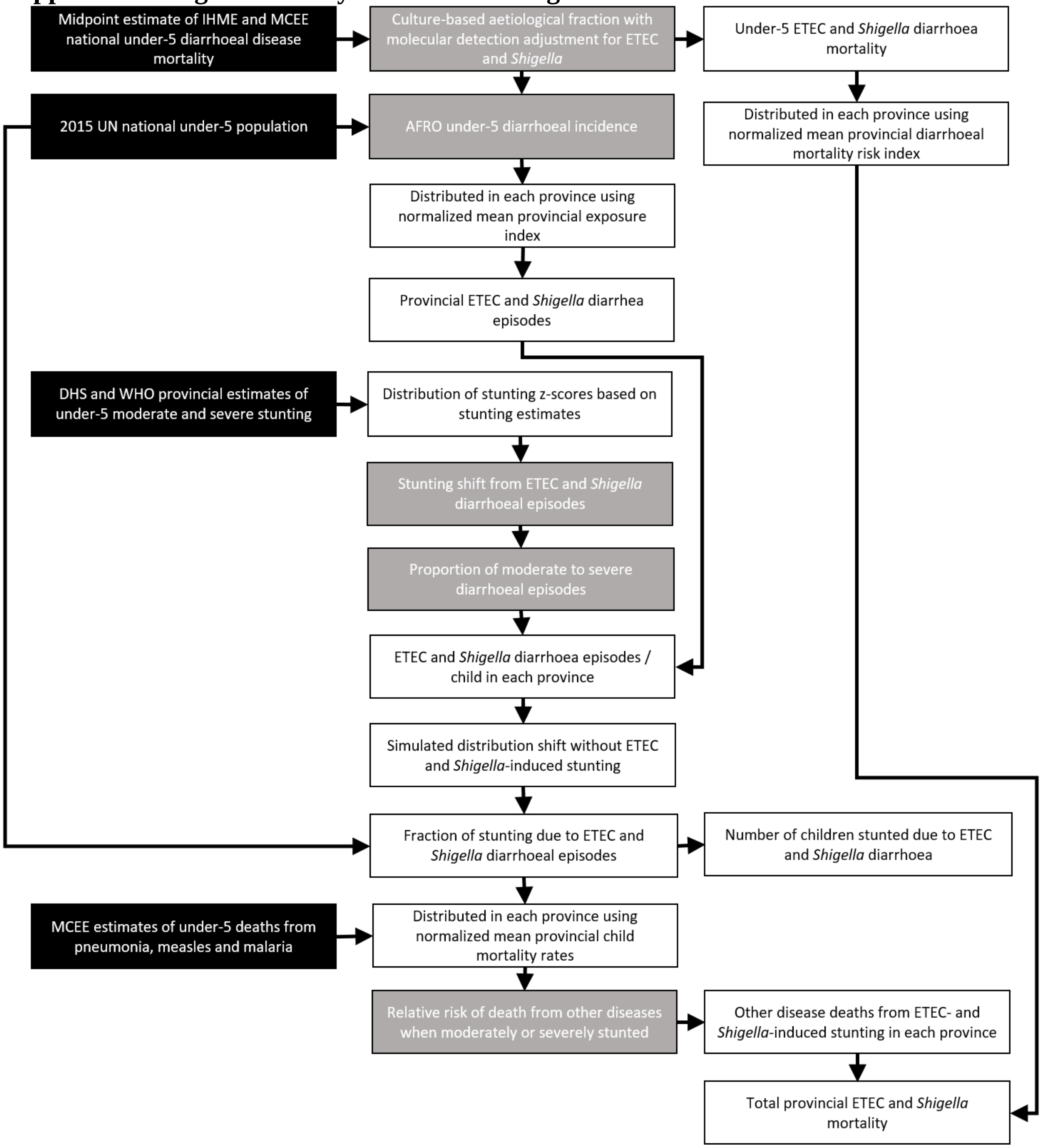

Supplemental Figure 3. Tornado plots of key input variables on ETEC and Shigella morbidity and mortality estimates.

HAZ shift is the fraction of ETEC and Shigella-attributable episodes that result in a shift of HAZ scores for children. HAZ: height-for-age Z-score. MSD: moderate-to-severe diarrhoea.

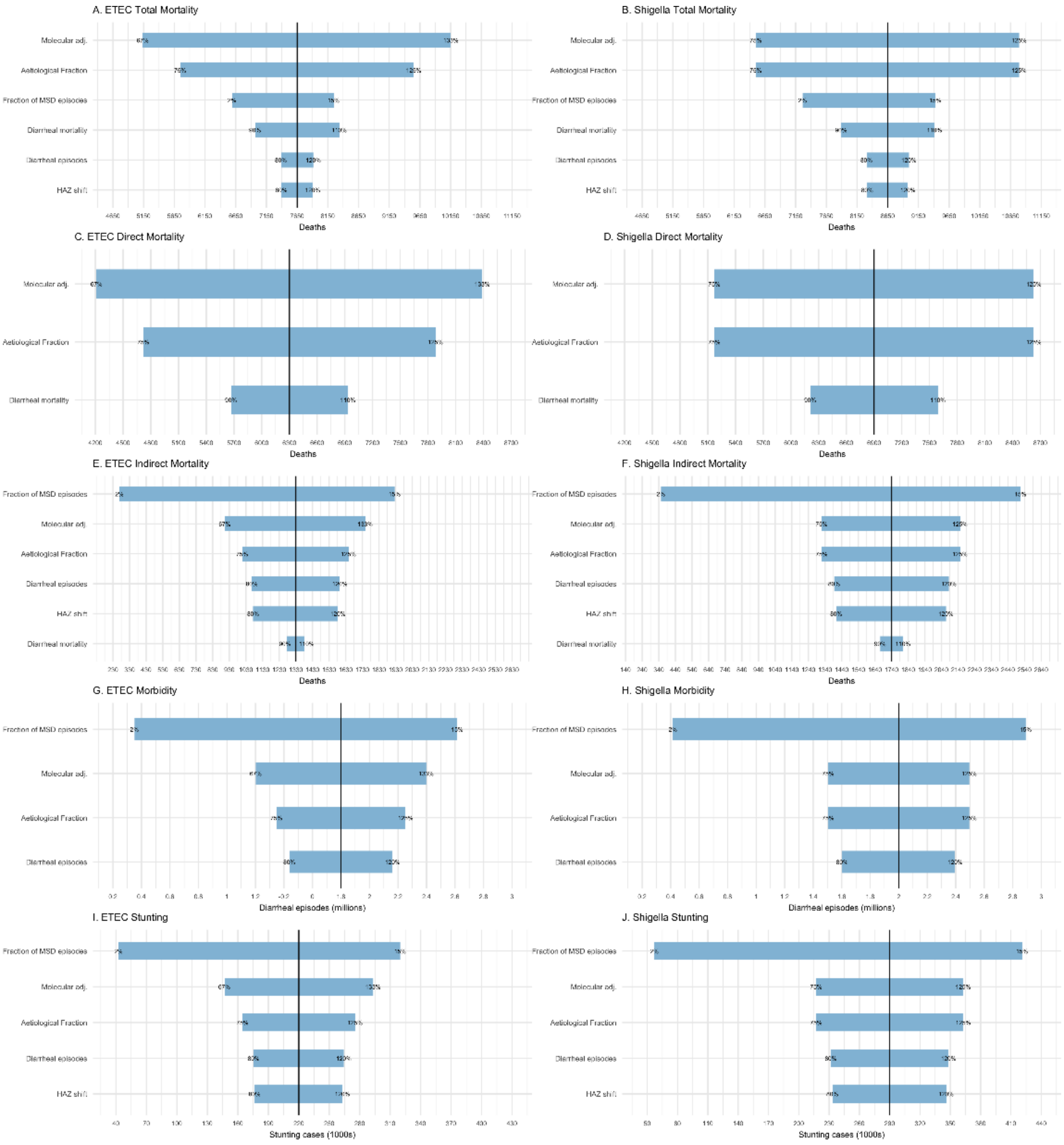

Supplementary Table 1. Relative risk values from literature and Demographic and Health survey (DHS) data used to calculate exposure (A) and susceptibility (B) indices.

A. Exposure Index

Input	RR Value	Description	Reference
**Water access RR**		**DHS Household File**	**Country-specific DHS**
Unimproved	1.00	"Dug well: Unprotected Well", "Water from Spring: Unprotected Spring", "Tanker Truck", "Cart with Small Tank", "Surface Water (River/Dam/Lake/Pond/Stream/Canal/Irrigation Channel)", "Bottled Water"	HV201
Off-plot improved	0.89	"Piped Water to Neighbour", "Public Tap/Standpipe", "Tube Well or Borehole", "Dug Well: Protected Well", "Water from Spring: Protected Spring" and "Rainwater"	HV201
On-plot improved	0.77	"Piped into Dwelling" or "Piped to Yard/Plot", and "On Premises" improved water source	HV201, HV235
**Sanitation access RR**		**DHS Household File**	**Country-specific DHS**
Unimproved and shared	1.00	"Flush or pour flush toilet: Flush to somewhere else", "Pit Latrine: without slab/open pit", "Bucket Toilet", "Hanging Toilet/Hanging Latrine", "No Facility/Bush/Field"	HV205
Improved and unshared (excluding sewered house connection)	0.84	"Flush or pour flush toilet: Flush to septic tank/pit latrine", Flush or pour flush: don't know where", "Pit Latrine: VIP/with slab", "Composting toilet"	HV205
Improved, unshared (with sewered house connection)	0.31	"Flush or pour flush toilet flush to piped sewer system"	HV205, HV225

B. Susceptibility index

Input	RR Value	Description	Reference
**Child underweight**		**DHS Child File**	**Country-specific DHS**
No risk	1.00	WFA z-score > -1 standard deviations (SD) from the mean	Caulfield et al. 2004
Mild risk	2.32	WFA z-score -1 to -2 SD from the mean	
Moderate risk	5.39	WFA z-score -2 to -3 SD from the mean	
High risk	12.50	WFA z-score < -3 SD from the mean	
**Oral rehydration treatment**		**DHS Child File**	**Country-specific DHS**
Probability of ORT	-	Imputed value from logistic regression of children who received "Recommended home solution" or "Oral rehydration solution"	H13 and H14 or equivalent
Diarrhoeal mortality risk reduction from receiving ORT	0.07	Protective, reduces risk of mortality by 93%	Munoz et al. 2010
**Vitamin A dose**		**DHS Child File**	**Country-specific DHS**
Received vitamin A dose	-	Vaccine card or mother' recall	H34
Diarrhoeal mortality risk reduction from receiving vitamin A	0.72	Protective, reduces risk of mortality by 28%	Imdad et al. 2011

Supplementary Table 2. Exposure scenarios and assigned relative risks from literature estimates.

Scenario		Relative risk water	Relative risk sanitation	Combined relative risks
1	No improved water access, no improved sanitation access	1.00	1.00	1.00
2	Improved off-plot water access, no improved sanitation access	0.89	1.00	0.89
3	Improved on-plot water access, no improved sanitation access	0.77	1.00	0.77
4	No improved water access, improved sanitation access	1.00	0.84	0.84
5	Improved off-plot water access, improved sanitation access	0.89	0.84	0.75
6	Improved on-plot water access, improved sanitation access	0.77	0.84	0.65
7	No improved water access, sewer connection	1.00	0.31	0.31
8	Improved off-plot water access, sewer connection	0.89	0.31	0.28
9	Improved on-plot water access, sewer connection	0.77	0.31	0.24

Supplementary Table 3. **Key assumptions and inputs in calculating ETEC and *Shigella* burden.**
Ranges for estimates reflect ranges of values varied for uncertainty analysis, unless otherwise noted in the table.

Key assumptions and inputs	Values	Range	Source	Reference
Population estimates for children under 5 years of age	Varies by province and country	Not included in uncertainty analysis	Malaria Atlas Project	22
Diarrhoeal mortality estimates	Differ in overall deaths and per country	± 10%; Triangular*	Midpoint estimate between 2016 MCEE and GBD	24,25
Aetiological fraction attributed to ETEC for children under 5 by WHO region	0.075	± 25%; Triangular*	Based on systematic review and updated with GEMS molecular re-analysis estimates	16,26
Aetiological fraction attributed to *Shigella* for children under 5 by WHO region	0.082	± 25%; Triangular*	Based on systematic review and updated with GEMS molecular re-analysis estimates	16,26
ETEC molecular adjustment	1·5	±0·5; Triangular*	GEMS re-analysis study	16
Shigella molecular adjustment	2·0	±0·5; Triangular*	GEMS re-analysis study	16
U5C all-cause diarrhoeal episodes	3·3 episodes / child-year	± 10%; Triangular*	Fischer-Walker et al.	27
Fraction of diarrhoeal episodes that are moderate-to-severe	0·10	0.02 – 0.15; Triangular*	GEMS Study and DHS	17,28
Stunting attributed to ETEC episodes	0·068 shift in HAZ	± 20%; Triangular*	GEMS estimate Table 6	17
Stunting attributed to *Shigella* episodes	0·082 shift in HAZ	± 20%; Triangular*	GEMS estimate Table 6	17

Supplementary Table 4. National and subnational ETEC morbidity and mortality estimates for under-five children in 2016 for 11 eastern and central African countries.

National estimates are shown in bold. Subnational estimates are in plain text. Estimates are followed by their uncertainty intervals, shown in parentheses. Averages of regional estimates are approximately equal to national estimates (see Methods). ETEC: enterotoxigenic Escherichia coli; DRC: Democratic Republic of Congo; MSD: moderate-to-severe diarrhoea; MSS: moderate and severe stunting; U5C: under-five child.

**Country** Province	**ETEC MSD Episodes**	**ETEC MSD Episodes per 100,000 U5C/year**	**ETEC-attributable MSS Cases**	**ETEC-attributable MSS Cases per 100,000 U5C/year**	**Direct ETEC Deaths**	**Direct Mortality Rate per 100,000 U5C/year**	**Indirect ETEC Deaths**	**Indirect Mortality Rate per 100,000 U5C/year**	**Total ETEC Deaths**	**Total ETEC Mortality Rate per 100,000 U5C/year**
**Burundi**	**53,715 (17,680–82,777)**	**2,563 (843–3,949)**	**8,366 (2,731–13,115)**	**399 (130–626)**	**394 (271–536)**	**18.8 (12.9–25.6)**	**82 (27–127)**	**3.9 (1.3–6.1)**	**476 (317–642)**	**22.7 (15.1–30.6)**
Bubanza	2,290 (754–3,529)	2,631 (866–4,055)	350 (114–549)	403 (131–631)	15 (10–20)	17.3 (11.9–23.5)	3 (1–5)	3.6 (1.2–5.6)	18 (12–25)	20.9 (13.9–28.2)
Bujumbura Mairie	2,190 (721–3,375)	2,383 (784–3,673)	220 (72–345)	240 (78–376)	11 (8–15)	11.9 (8.2–16.2)	1 (0–2)	1.3 (0.4–2.0)	12 (8–16)	13.2 (9.0–17.8)
Bujumbura Rural	4,493 (1,479–6,924)	2,578 (849–3,973)	694 (227–1,088)	398 (130–625)	35 (24–47)	19.9 (13.7–27.1)	5 (2–8)	3.1 (1.0–4.8)	40 (27–54)	23.0 (15.5–31.0)
Bururi	2,242 (738–3,455)	2,465 (811–3,799)	343 (112–537)	377 (123–590)	15 (11–21)	16.9 (11.6–23.0)	2 (1–4)	2.5 (0.8–3.9)	18 (12–24)	19.4 (13.1–26.2)
Cankuzo	1,522 (501–2,345)	2,614 (860–4,028)	252 (82–396)	434 (142–680)	11 (7–15)	18.4 (12.6–25.1)	2 (1–4)	4.3 (1.4–6.6)	13 (9–18)	22.7 (15.1–30.7)
Cibitoke	3,011 (991–4,640)	2,545 (838–3,922)	501 (164–785)	423 (138–664)	17 (12–24)	14.7 (10.1–20.0)	5 (2–8)	4.1 (1.4–6.4)	22 (15–30)	18.9 (12.4–25.5)
Gitega	5,218 (1,717–8,041)	2,580 (849–3,975)	743 (243–1,165)	367 (120–576)	34 (23–46)	16.7 (11.4–22.7)	7 (2–12)	3.7 (1.2–5.7)	41 (27–55)	20.3 (13.5–27.4)
Karuzi	2,621 (863–4,039)	2,369 (780–3,651)	491 (160–770)	444 (145–696)	22 (15–29)	19.4 (13.3–26.5)	5 (2–7)	4.1 (1.4–6.4)	26 (17–35)	23.6 (15.7–31.8)
Kayanza	3,838 (1,263–5,914)	2,554 (841–3,936)	691 (226–1,083)	460 (150–721)	29 (20–39)	19.3 (13.2–26.2)	6 (2–10)	4.2 (1.4–6.5)	35 (23–47)	23.4 (15.6–31.6)
Kirundo	4,211 (1,386–6,490)	2,696 (888–4,155)	661 (216–1,037)	423 (138–664)	34 (24–47)	22.0 (15.1–29.9)	10 (3–15)	6.2 (2.0–9.6)	44 (29–59)	28.2 (18.5–38.1)
Makamba	2,157 (710–3,324)	2,430 (800–3,744)	311 (101–487)	350 (114–549)	13 (9–18)	15.2 (10.4–20.7)	2 (1–3)	2.1 (0.7–3.3)	15 (10–21)	17.3 (11.7–23.4)

**Country** Province	**ETEC MSD Episodes**	**ETEC MSD Episodes per 100,000 U5C/year**	**ETEC-attributable MSS Cases**	**ETEC-attributable MSS Cases per 100,000 U5C/year**	**Direct ETEC Deaths**	**Direct Mortality Rate per 100,000 U5C/year**	**Indirect ETEC Deaths**	**Indirect Mortality Rate per 100,000 U5C/year**	**Total ETEC Deaths**	**Total ETEC Mortality Rate per 100,000 U5C/ year**
Muramvya	1,834 (604–2,826)	2,413 (794–3,719)	329 (108–517)	434 (142–680)	18 (12–25)	23.9 (16.4–32.6)	4 (1–5)	4.6 (1.5–7.1)	22 (14–29)	28.5 (19.0–38.5)
Muyinga	4,458 (1,467–6,870)	2,653 (873–4,089)	657 (215–1,031)	391 (128–614)	34 (23–47)	20.4 (14.0–27.7)	9 (3–14)	5.2 (1.7–8.1)	43 (28–58)	25.6 (16.9–34.6)
Mwaro	1,850 (609–2,851)	2,461 (810–3,792)	299 (98–469)	398 (130–623)	17 (12–23)	22.6 (15.5–30.8)	2 (1–4)	3.3 (1.1–5.0)	19 (13–26)	25.9 (17.5–34.9)
Ngozi	4,727 (1,556–7,284)	2,681 (883–4,132)	746 (244–1,170)	423 (138–664)	38 (26–51)	21.3 (14.6–29.0)	9 (3–14)	5.2 (1.7–8.1)	47 (31–63)	26.5 (17.6–35.8)
Rumonge	1,909 (628–2,941)	2,496 (821–3,846)	322 (105–505)	421 (138–660)	14 (10–19)	18.5 (12.7–25.2)	3 (1–4)	3.7 (1.2–5.8)	17 (11–23)	22.2 (14.8–29.9)
Rutana	2,349 (773–3,620)	2,629 (865–4,052)	338 (111–531)	379 (124–594)	18 (12–24)	19.7 (13.5–26.7)	3 (1–4)	3.1 (1.0–4.8)	20 (14–27)	22.7 (15.3–30.7)
Ruyigi	2,795 (920–4,308)	2,636 (867–4,061)	415 (136–651)	392 (128–614)	19 (13–26)	18.3 (12.5–24.8)	3 (1–5)	3.2 (1.1–5.0)	23 (15–31)	21.5 (14.4–29.0)
**DRC**	**363,320 (119,583–559,891)**	**2,582 (850– 3,979)**	**52,115 (17,032–81,678)**	**370 (121– 580)**	**1,977 (1,357–2,690)**	**14.0 (9.6– 19.1)**	**528 (174–819)**	**3.8 (1.2– 5.8)**	**2,505 (1,653–3,384)**	**17.8 (11.7– 24.1)**
Bandundu	40,037 (13,178– 61,698)	2,655 (874– 4,092)	5,510 (1,801–8,635)	365 (119– 573)	195 (134– 266)	13.0 (8.9– 17.6)	44 (15– 69)	2.9 (1.0– 4.6)	240 (159– 324)	15.9 (10.6– 21.5)
Bas Congo	14,638 (4,818– 22,558)	2,597 (855– 4,002)	2,339 (764– 3,666)	415 (136– 650)	65 (45– 89)	11.6 (8.0– 15.8)	24 (8– 37)	4.2 (1.4– 6.6)	89 (58– 121)	15.8 (10.2– 21.5)
Equateur	38,077 (12,533– 58,678)	2,714 (893– 4,182)	4,830 (1,578–7,569)	344 (112– 539)	229 (157– 312)	16.3 (11.2– 22.2)	57 (19– 88)	4.0 (1.3– 6.3)	286 (189– 386)	20.4 (13.5– 27.5)
Kasai Occidental	30,925 (10,179– 47,657)	2,727 (898– 4,203)	5,170 (1,689–8,104)	456 (149– 715)	177 (122– 241)	15.6 (10.7– 21.3)	65 (21– 101)	5.8 (1.9– 8.9)	243 (157– 329)	21.4 (13.8– 29.0)
Kasai Oriental	39,836 (13,112– 61,389)	2,676 (881– 4,123)	5,933 (1,938–9,299)	398 (130– 625)	240 (165– 326)	16.1 (11.1– 21.9)	64 (21– 99)	4.3 (1.4– 6.7)	304 (201– 411)	20.4 (13.5– 27.6)
Katanga	46,693 (15,368– 71,956)	2,622 (863– 4,040)	7,014	394 (129– 617)	257 (177– 350)	14.5 (9.9– 19.7)	73 (24– 113)	4.1 (1.3– 6.4)	330 (217– 447)	18.5 (12.2– 25.1)

Country<br>Province	**ETEC MSD Episodes**	**ETEC MSD Episodes per 100,000 U5C/year**	**ETEC-attributable MSS Cases**	**ETEC-attributable MSS Cases per 100,000 U5C/year**	**Direct ETEC Deaths**	**Direct Mortality Rate per 100,000 U5C/year**	**Indirect ETEC Deaths**	**Indirect Mortality Rate per 100,000 U5C/year**	**Total ETEC Deaths**	**Total ETEC Mortality Rate per 100,000 U5C/ year**
			(2,292– 10,994)							
Kinshasa	33,823 (11,132– 52,122)	2,336 (769– 3,599)	2,266 (740– 3,550)	156 (51– 245)	95 (65– 129)	6.6 (4.5– 8.9)	8 (3– 12)	0.5 (0.2– 0.8)	103 (70– 139)	7.1 (4.8– 9.6)
Maniema	9,329 (3,071– 14,376)	2,497 (822– 3,848)	1,316 (430– 2,063)	352 (115– 552)	53 (37– 73)	14.3 (9.8– 19.4)	11 (4– 18)	3.1 (1.0– 4.7)	65 (43– 87)	17.3 (11.5– 23.3)
Nord Kivu	23,645 (7,783– 36,439)	2,450 (806– 3,775)	4,136 (1,352– 6,482)	428 (140– 672)	126 (87– 172)	13.1 (9.0– 17.8)	31 (10– 49)	3.2 (1.1– 5.0)	157 (104– 213)	16.3 (10.8– 22.0)
Orientale	68,021 (22,388– 104,823)	2,520 (830– 3,884)	10,429 (3,408– 16,345)	386 (126– 606)	418 (287– 569)	15.5 (10.6– 21.1)	108 (36– 167)	4.0 (1.3– 6.2)	526 (348– 711)	19.5 (12.9– 26.3)
Sud Kivu	18,296 (6,022– 28,194)	2,588 (852– 3,989)	3,171 (1,036– 4,970)	449 (147– 703)	120 (82– 163)	16.9 (11.6– 23.0)	43 (14– 66)	6.0 (2.0– 9.3)	162 (105– 220)	22.9 (14.9– 31.1)
**Ethiopia**	**383,244 (126,141– 590,595)**	**2,593 (853– 3,995)**	**50,651 (16,553– 79,383)**	**343 (112– 537)**	**1,141 (784– 1,553)**	**7.7 (5.3– 10.5)**	**251 (83– 390)**	**1.7 (0.6– 2.6)**	**1,393 (925– 1,879)**	**9.4 (6.3– 12.7)**
Addis Ababa	11,927 (3,926– 18,379)	1,849 (609– 2,849)	921 (301– 1,443)	143 (47– 224)	9 (6– 13)	1.5 (1.0– 2.0)	1 (0– 2)	0.2 (0.1– 0.3)	11 (7– 14)	1.6 (1.1– 2.2)
Afar	7,846 (2,582– 12,091)	2,615 (861– 4,030)	1,159 (379– 1,817)	386 (126– 606)	32 (22– 43)	10.5 (7.2– 14.3)	9 (3– 13)	2.9 (1.0– 4.5)	40 (26– 54)	13.4 (8.8– 18.1)
Amhara	99,411 (32,720– 153,197)	2,642 (870– 4,072)	14,253 (4,656– 22,340)	379 (124– 594)	339 (233– 461)	9.0 (6.2– 12.3)	75 (25– 117)	2.0 (0.7– 3.1)	414 (275– 559)	11.0 (7.3– 14.8)
Benishangul-Gumuz	3,933 (1,295– 6,061)	2,582 (850– 3,978)	589 (192– 923)	386 (126– 606)	10 (7– 13)	6.3 (4.3– 8.6)	3 (1– 5)	2.1 (0.7– 3.2)	13 (8– 17)	8.4 (5.5– 11.3)
Dire Dawa	1,916 (631– 2,952)	2,297 (756– 3,540)	311 (102– 487)	373 (122– 584)	4 (3– 6)	5.1 (3.5– 7.0)	1 (0– 2)	1.7 (0.6– 2.7)	6 (4– 8)	6.8 (4.4– 9.3)
Gambela	1,575 (518– 2,427)	2,522 (830– 3,886)	150 (49– 235)	240 (78– 376)	4 (3– 5)	6.1 (4.2– 8.3)	1 (0– 1)	1.0 (0.3– 1.5)	4 (3– 6)	7.0 (4.7– 9.5)
Harari	1,024 (337– 1,578)	2,408 (793– 3,711)	134 (44– 210)	316 (103– 495)	2 (2– 3)	5.1 (3.5– 7.0)	0 (0– 1)	1.2 (0.4– 1.8)	3 (2– 4)	6.3 (4.2– 8.5)
Oromia	137,350	2,641	17,434	335	438	8.4	86	1.7	524	10.1

Country Province	ETEC MSD Episodes	ETEC MSD Episodes per 100,000 U5C/year	ETEC-attributable MSS Cases	ETEC-attributable MSS Cases per 100,000 U5C/year	Direct ETEC Deaths	Direct Mortality Rate per 100,000 U5C/year	Indirect ETEC Deaths	Indirect Mortality Rate per 100,000 U5C/year	Total ETEC Deaths	Total ETEC Mortality Rate per 100,000 U5C/year
	(45,207– 211,662)	(869– 4,070)	(5,697– 27,322)	(110– 525)	(301– 596)	(5.8– 11.5)	(28– 133)	(0.5– 2.6)	(349– 706)	(6.7– 13.6)
SNNPR	75,323 (24,792– 116,075)	2,640 (869– 4,068)	10,751 (3,513– 16,849)	377 (123– 590)	196 (135– 267)	6.9 (4.7– 9.4)	56 (19– 87)	2.0 (0.6– 3.1)	252 (166– 341)	8.8 (5.8– 12.0)
Somali	21,060 (6,932– 32,454)	2,597 (855– 4,002)	2,316 (756– 3,629)	286 (93– 447)	58 (40– 79)	7.1 (4.9– 9.7)	11 (4– 17)	1.4 (0.4– 2.1)	69 (46– 93)	8.5 (5.7– 11.4)
Tigray	21,880 (7,202– 33,718)	2,517 (828– 3,878)	2,634 (860– 4,128)	303 (99– 475)	50 (34– 68)	5.7 (3.9– 7.8)	8 (2– 12)	0.9 (0.3– 1.4)	57 (39– 77)	6.6 (4.4– 8.9)
**Kenya**	**189,499 (62,372– 292,027)**	**2,648 (872– 4,081)**	**16,998 (5,552– 26,637)**	**238 (78– 372)**	**491 (337– 668)**	**6.9 (4.7– 9.3)**	**52 (17– 81)**	**0.7 (0.2– 1.1)**	**543 (368– 732)**	**7.6 (5.1– 10.2)**
Central	18,480 (6,082– 28,478)	2,428 (799– 3,742)	1,328 (434– 2,081)	174 (57– 273)	38 (26– 51)	4.9 (3.4– 6.7)	3 (1– 4)	0.4 (0.1– 0.6)	40 (28– 55)	5.3 (3.6– 7.2)
Coast	15,697 (5,166– 24,190)	2,700 (889– 4,161)	1,627 (531– 2,549)	280 (91– 438)	37 (26– 51)	6.4 (4.4– 8.7)	5 (2– 8)	0.9 (0.3– 1.4)	43 (29– 57)	7.3 (4.9– 9.9)
Eastern	26,913 (8,858– 41,474)	2,707 (891– 4,171)	2,580 (843– 4,043)	259 (85– 407)	81 (56– 111)	8.2 (5.6– 11.2)	7 (2– 11)	0.7 (0.2– 1.1)	89 (60– 120)	8.9 (6.1– 12.0)
Nairobi	11,495 (3,783– 17,714)	2,261 (744– 3,484)	838 (274– 1,313)	165 (54– 258)	20 (14– 27)	3.9 (2.7– 5.4)	2 (1– 3)	0.4 (0.1– 0.6)	22 (15– 30)	4.4 (2.9– 5.9)
North Eastern	10,987 (3,616– 16,932)	2,781 (915– 4,285)	957 (313– 1,499)	242 (79– 379)	31 (22– 43)	8.0 (5.5– 10.8)	3 (1– 5)	0.8 (0.3– 1.3)	35 (24– 47)	8.8 (6.0– 11.9)
Nyanza	30,571 (10,062– 47,112)	2,725 (897– 4,199)	2,531 (827– 3,966)	226 (74– 353)	73 (50– 100)	6.5 (4.5– 8.9)	10 (3– 15)	0.9 (0.3– 1.3)	83 (56– 112)	7.4 (5.0– 10.0)
Rift Valley	50,120 (16,497– 77,238)	2,673 (880– 4,119)	4,951 (1,618– 7,759)	264 (86– 414)	147 (101– 201)	7.9 (5.4– 10.7)	15 (5– 23)	0.8 (0.3– 1.2)	162 (110– 219)	8.6 (5.9– 11.7)
Western	25,236 (8,306– 38,890)	2,749 (905– 4,236)	2,188 (715– 3,428)	238 (78– 373)	62 (43– 85)	6.8 (4.7– 9.3)	7 (2– 11)	0.8 (0.2– 1.2)	69 (47– 94)	7.6 (5.1– 10.2)
**Malawi**	**76,202 (25,081– 117,430)**	**2,584 (851– 3,982)**	**8,560 (2,797– 13,416)**	**290 (95– 455)**	**222 (152– 302)**	**7.5 (5.2– 10.2)**	**37 (12– 57)**	**1.2 (0.4– 1.9)**	**259 (173– 349)**	**8.8 (5.9– 11.8)**

**Country** Province	**ETEC MSD Episodes**	**ETEC MSD Episodes per 100,000 U5C/year**	**ETEC-attributable MSS Cases**	**ETEC-attributable MSS Cases per 100,000 U5C/year**	**Direct ETEC Deaths**	**Direct Mortality Rate per 100,000 U5C/year**	**Indirect ETEC Deaths**	**Indirect Mortality Rate per 100,000 U5C/year**	**Total ETEC Deaths**	**Total ETEC Mortality Rate per 100,000 U5C/ year**
Central	32,009 (10,536– 49,328)	2,577 (848– 3,972)	3,549 (1,160– 5,563)	286 (93– 448)	91 (63– 124)	7.4 (5.1– 10.0)	15 (5– 24)	1.2 (0.4– 1.9)	107 (72– 144)	8.6 (5.8– 11.6)
Northern	9,284 (3,056– 14,307)	2,571 (846– 3,962)	1,015 (332– 1,591)	281 (92– 441)	32 (22– 43)	8.8 (6.0– 11.9)	4 (1– 6)	1.1 (0.3– 1.6)	36 (24– 48)	9.8 (6.6– 13.3)
Southern	34,909 (11,490– 53,796)	2,594 (854– 3,998)	3,995 (1,306– 6,262)	297 (97– 465)	99 (68– 134)	7.3 (5.0– 10.0)	17 (6– 27)	1.3 (0.4– 2.0)	116 (78– 157)	8.6 (5.8– 11.7)
**Mozambique**	**123,222 (40,557– 189,890)**	**2,580 (849– 3,975)**	**17,010 (5,559– 26,660)**	**356 (116– 558)**	**368 (253– 501)**	**7.7 (5.3– 10.5)**	**107 (35– 166)**	**2.2 (0.7– 3.5)**	**475 (312– 642)**	**9.9 (6.5– 13.4)**
Cabo Delgado	9,604 (3,161– 14,800)	2,716 (894– 4,186)	1,489 (486– 2,334)	421 (138– 660)	56 (38– 76)	15.8 (10.9– 21.6)	14 (5– 21)	3.9 (1.3– 6.1)	70 (46– 94)	19.8 (13.1– 26.7)
Gaza	5,659 (1,863– 8,721)	2,465 (811– 3,799)	550 (180– 863)	240 (78– 376)	8 (6– 11)	3.7 (2.5– 5.0)	2 (1– 4)	1.1 (0.3– 1.6)	11 (7– 15)	4.7 (3.1– 6.4)
Inhambane	7,333 (2,414– 11,301)	2,554 (841– 3,936)	906 (296– 1,420)	316 (103– 495)	10 (7– 13)	3.4 (2.4– 4.7)	3 (1– 4)	1.0 (0.3– 1.5)	13 (8– 17)	4.4 (2.9– 6.0)
Manica	9,012 (2,966– 13,887)	2,489 (819– 3,836)	1,249 (408– 1,957)	345 (113– 541)	17 (11– 23)	4.6 (3.1– 6.2)	7 (2– 11)	1.9 (0.6– 3.0)	24 (15– 32)	6.5 (4.2– 8.9)
Maputo	11,673 (3,842– 17,989)	2,187 (720– 3,371)	1,136 (371– 1,780)	213 (70– 334)	22 (15– 30)	4.1 (2.8– 5.6)	4 (1– 6)	0.7 (0.2– 1.1)	26 (17– 35)	4.9 (3.3– 6.6)
Nampula	24,667 (8,119– 38,012)	2,632 (866– 4,057)	3,932 (1,285– 6,163)	420 (137– 658)	70 (48– 95)	7.5 (5.1– 10.2)	19 (6– 30)	2.1 (0.7– 3.2)	89 (59– 121)	9.5 (6.3– 12.9)
Niassa	7,888 (2,596– 12,156)	2,566 (845– 3,954)	1,225 (400– 1,920)	398 (130– 625)	10 (7– 13)	3.2 (2.2– 4.4)	6 (2– 10)	2.0 (0.7– 3.1)	16 (10– 22)	5.2 (3.2– 7.2)
Sofala	9,832 (3,236– 15,151)	2,560 (843– 3,945)	1,249 (408– 1,958)	325 (106– 510)	17 (12– 23)	4.5 (3.1– 6.1)	6 (2– 10)	1.6 (0.5– 2.5)	23 (15– 32)	6.1 (3.9– 8.2)
Tete	12,146 (3,998– 18,717)	2,645 (871– 4,076)	1,639 (536– 2,569)	357 (117– 559)	54 (37– 74)	11.8 (8.1– 16.0)	13 (4– 20)	2.8 (0.9– 4.3)	67 (44– 90)	14.5 (9.6– 19.7)
Zambezia	25,408	2,753	3,634	394	104	11.2	33	3.5	136	14.7

**Country** Province	**ETEC MSD Episodes**	**ETEC MSD Episodes per 100,000 U5C/year**	**ETEC-attributable MSS Cases**	**ETEC-attributable MSS Cases per 100,000 U5C/year**	**Direct ETEC Deaths**	**Direct Mortality Rate per 100,000 U5C/year**	**Indirect ETEC Deaths**	**Indirect Mortality Rate per 100,000 U5C/year**	**Total ETEC Deaths**	**Total ETEC Mortality Rate per 100,000 U5C/ year**
	(8,363– 39,154)	(906– 4,243)	(1,188– 5,696)	(129– 617)	(71– 141)	(7.7– 15.3)	(11– 50)	(1.2– 5.5)	(89– 184)	(9.6– 20.0)
**Rwanda**	**46,257** **(15,225– 71,284)**	**2,590** **(852– 3,991)**	**5,791** **(1,893– 9,077)**	**324** **(106– 508)**	**94** **(65– 129)**	**5.3** **(3.6– 7.2)**	**19** **(6– 30)**	**1.1** **(0.4– 1.7)**	**114** **(76– 153)**	**6.4** **(4.2– 8.6)**
Eastern	11,099 (3,653– 17,104)	2,611 (859– 4,023)	1,355 (443– 2,124)	319 (104– 500)	23 (16– 31)	5.4 (3.7– 7.4)	5 (2– 8)	1.2 (0.4– 1.9)	28 (19– 38)	6.6 (4.4– 9.0)
Kigali City	4,568 (1,503– 7,039)	2,481 (816– 3,823)	395 (129– 619)	214 (70– 336)	8 (5– 10)	4.1 (2.8– 5.6)	1 (0– 1)	0.3 (0.1– 0.5)	8 (6– 11)	4.5 (3.1– 6.1)
Northern	7,081 (2,331– 10,913)	2,532 (833– 3,902)	930 (304– 1,458)	333 (109– 521)	13 (9– 18)	4.6 (3.2– 6.3)	3 (1– 4)	1.0 (0.3– 1.6)	16 (11– 21)	5.7 (3.8– 7.6)
Southern	10,731 (3,532– 16,537)	2,641 (869– 4,071)	1,352 (442– 2,118)	333 (109– 521)	24 (17– 33)	6.0 (4.1– 8.1)	5 (2– 7)	1.2 (0.4– 1.8)	29 (19– 39)	7.1 (4.8– 9.6)
Western	12,778 (4,206– 19,692)	2,603 (857– 4,011)	1,759 (575– 2,758)	358 (117– 562)	26 (18– 36)	5.4 (3.7– 7.3)	6 (2– 9)	1.2 (0.4– 1.9)	32 (21– 44)	6.6 (4.4– 8.9)
**Tanzania**	**246,491** **(81,130– 379,853)**	**2,642** **(870– 4,071)**	**27,556** **(9,005– 43,188)**	**295** **(97– 463)**	**597** **(410– 812)**	**6.4** **(4.4– 8.7)**	**100** **(33– 155)**	**1.1** **(0.4– 1.7)**	**697** **(467– 940)**	**7.5** **(5.0– 10.1)**
Central	30,683 (10,099– 47,284)	2,706 (891– 4,169)	3,026 (989– 4,743)	267 (87– 418)	88 (60– 119)	7.7 (5.3– 10.5)	9 (3– 15)	0.8 (0.3– 1.3)	97 (66– 131)	8.6 (5.8– 11.6)
Eastern	32,959 (10,848– 50,792)	2,485 (818– 3,829)	2,992 (977– 4,689)	226 (74– 353)	71 (49– 97)	5.4 (3.7– 7.3)	9 (3– 15)	0.7 (0.2– 1.1)	81 (54– 109)	6.1 (4.1– 8.2)
Lake	68,530 (22,556– 105,608)	2,729 (898– 4,205)	7,890 (2,578– 12,365)	314 (103– 492)	162 (112– 221)	6.5 (4.4– 8.8)	34 (11– 52)	1.3 (0.4– 2.1)	196 (130– 264)	7.8 (5.2– 10.5)
Northern	28,067 (9,238– 43,252)	2,458 (809– 3,787)	3,715 (1,214– 5,821)	325 (106– 510)	63 (44– 86)	5.6 (3.8– 7.6)	12 (4– 19)	1.1 (0.3– 1.6)	76 (50– 102)	6.6 (4.4– 8.9)
Southern	12,022 (3,957– 18,526)	2,673 (880– 4,120)	1,285 (420– 2,014)	286 (93– 448)	25 (17– 34)	5.5 (3.8– 7.5)	4 (1– 7)	0.9 (0.3– 1.4)	29 (20– 39)	6.5 (4.3– 8.7)

Country<br>Province	ETEC MSD Episodes	ETEC MSD Episodes per 100,000 U5C/year	ETEC-attributable MSS Cases	ETEC-attributable MSS Cases per 100,000 U5C/year	Direct ETEC Deaths	Direct Mortality Rate per 100,000 U5C/year	Indirect ETEC Deaths	Indirect Mortality Rate per 100,000 U5C/year	Total ETEC Deaths	Total ETEC Mortality Rate per 100,000 U5C/year
Southern Highlands	43,011 (14,157– 66,282)	2,698 (888– 4,158)	5,400 (1,764– 8,463)	339 (111– 531)	105 (72– 142)	6.6 (4.5– 8.9)	20 (7– 31)	1.3 (0.4– 1.9)	124 (83– 168)	7.8 (5.2– 10.5)
Western	26,213 (8,628– 40,396)	2,765 (910– 4,261)	2,745 (897– 4,301)	289 (95– 454)	72 (50– 98)	7.6 (5.2– 10.4)	10 (3– 16)	1.1 (0.4– 1.7)	83 (56– 111)	8.7 (5.9– 11.7)
Zanzibar	5,005 (1,647– 7,712)	2,239 (737– 3,450)	504 (165– 790)	226 (74– 353)	10 (7– 14)	4.5 (3.1– 6.2)	1 (0– 2)	0.5 (0.2– 0.8)	11 (8– 15)	5.0 (3.4– 6.8)
**Uganda**	**188,275 (61,969– 290,139)**	**2,565 (844– 3,953)**	**18,762 (6,131– 29,403)**	**256 (84– 401)**	**503 (345– 684)**	**6.8 (4.7– 9.3)**	**78 (26– 121)**	**1.1 (0.3– 1.7)**	**581 (391– 783)**	**7.9 (5.3– 10.7)**
Acholi	9,334 (3,072– 14,385)	2,610 (859– 4,023)	834 (273– 1,308)	233 (76– 366)	29 (20– 39)	8.0 (5.5– 10.9)	2 (1– 4)	0.6 (0.2– 1.0)	31 (21– 42)	8.6 (5.9– 11.7)
Ankole	13,243 (4,359– 20,408)	2,693 (886– 4,149)	1,376 (450– 2,156)	280 (91– 438)	47 (32– 64)	9.6 (6.6– 13.0)	7 (2– 10)	1.3 (0.4– 2.1)	54 (36– 72)	10.9 (7.4– 14.7)
Bugishu	6,965 (2,293– 10,734)	2,563 (844– 3,950)	854 (279– 1,338)	314 (103– 492)	25 (17– 33)	9.0 (6.2– 12.3)	4 (1– 6)	1.4 (0.5– 2.2)	28 (19– 38)	10.5 (7.0– 14.1)
Bukedi	8,903 (2,930– 13,720)	2,565 (844– 3,952)	738 (241– 1,156)	213 (69– 333)	20 (14– 28)	5.9 (4.0– 8.0)	3 (1– 5)	0.9 (0.3– 1.4)	24 (16– 32)	6.8 (4.6– 9.2)
Bunyoro	12,505 (4,116– 19,270)	2,611 (860– 4,024)	1,504 (492– 2,357)	314 (103– 492)	31 (22– 43)	6.5 (4.5– 8.9)	7 (2– 11)	1.5 (0.5– 2.4)	39 (26– 52)	8.1 (5.3– 10.9)
Busoga	20,428 (6,724– 31,481)	2,550 (839– 3,930)	2,149 (702– 3,368)	268 (88– 420)	47 (32– 64)	5.9 (4.0– 8.0)	10 (3– 15)	1.2 (0.4– 1.9)	57 (38– 77)	7.1 (4.7– 9.6)
Central 1	21,837 (7,187– 33,652)	2,518 (829– 3,880)	1,875 (613– 2,939)	216 (71– 339)	56 (39– 76)	6.5 (4.4– 8.8)	4 (1– 7)	0.5 (0.2– 0.8)	61 (41– 82)	7.0 (4.8– 9.4)
Central 2	19,699 (6,484– 30,357)	2,586 (851– 3,985)	1,903 (622– 2,982)	250 (82– 391)	50 (35– 69)	6.6 (4.5– 9.0)	7 (2– 11)	0.9 (0.3– 1.4)	57 (39– 77)	7.5 (5.1– 10.2)
Kampala	11,000 (3,621– 16,952)	2,250 (740– 3,467)	973 (318– 1,524)	199 (65– 312)	30 (20– 41)	6.1 (4.2– 8.3)	4 (1– 6)	0.9 (0.3– 1.3)	34 (23– 46)	6.9 (4.7– 9.4)
Karamoja	5,404 (1,779– 8,327)	2,596 (855– 4,001)	677 (221– 1,061)	325 (106– 510)	11 (8– 15)	5.4 (3.7– 7.3)	4 (1– 6)	1.8 (0.6– 2.8)	15 (10– 20)	7.2 (4.7– 9.8)
Kigezi	8,865 (2,918– 13,661)	2,626 (864– 4,046)	906 (296– 1,419)	268 (88– 420)	19 (13– 25)	5.5 (3.8– 7.5)	3 (1– 5)	1.0 (0.3– 1.6)	22 (15– 30)	6.5 (4.4– 8.8)

**Country** Province	**ETEC MSD Episodes**	**ETEC MSD Episodes per 100,000 U5C/year**	**ETEC-attributable MSS Cases**	**ETEC-attributable MSS Cases per 100,000 U5C/year**	**Direct ETEC Deaths**	**Direct Mortality Rate per 100,000 U5C/year**	**Indirect ETEC Deaths**	**Indirect Mortality Rate per 100,000 U5C/year**	**Total ETEC Deaths**	**Total ETEC Mortality Rate per 100,000 U5C/ year**
Lango	8,627 (2,840– 13,295)	2,598 (855– 4,004)	707 (231– 1,107)	213 (70– 334)	27 (18– 37)	8.1 (5.6– 11.0)	3 (1– 4)	0.8 (0.3– 1.2)	29 (20– 40)	8.9 (6.0– 12.0)
Teso	9,343 (3,075– 14,398)	2,550 (839– 3,929)	475 (155– 743)	130 (42– 203)	26 (18– 35)	7.0 (4.8– 9.6)	1 (0– 2)	0.4 (0.1– 0.6)	27 (19– 37)	7.4 (5.1– 10.0)
Tooro	15,931 (5,244– 24,551)	2,621 (863– 4,040)	1,946 (636– 3,050)	320 (105– 502)	40 (27– 54)	6.5 (4.5– 8.9)	9 (3– 14)	1.4 (0.5– 2.2)	49 (32– 65)	8.0 (5.3– 10.8)
West Nile	16,190 (5,340– 24,938)	2,603 (859– 4,010)	1,847 (601– 2,881)	297 (97– 463)	45 (31– 61)	7.2 (5.0– 9.8)	9 (3– 15)	1.5 (0.5– 2.4)	54 (36– 73)	8.7 (5.8– 11.8)
**Zambia**	**73,748 (24,273– 113,649)**	**2,550 (839– 3,930)**	**9,955 (3,254– 15,603)**	**344 (113– 540)**	**217 (149– 295)**	**7.5 (5.2– 10.2)**	**48 (16– 74)**	**1.7 (0.5– 2.6)**	**265 (176– 357)**	**9.2 (6.1– 12.4)**
Central	7,767 (2,557– 11,970)	2,586 (851– 3,985)	1,134 (370– 1,777)	377 (123– 592)	25 (17– 34)	8.2 (5.7– 11.2)	6 (2– 9)	2.0 (0.6– 3.0)	31 (20– 41)	10.2 (6.8– 13.8)
Copperbelt	9,776 (3,218– 15,066)	2,156 (710– 3,323)	1,424 (465– 2,232)	314 (103– 492)	27 (18– 37)	5.9 (4.1– 8.1)	5 (2– 8)	1.1 (0.4– 1.7)	32 (21– 43)	7.0 (4.7– 9.4)
Eastern	9,001 (2,962– 13,870)	2,635 (867– 4,060)	1,224 (400– 1,919)	358 (117– 562)	26 (18– 35)	7.6 (5.2– 10.3)	7 (2– 11)	2.2 (0.7– 3.4)	33 (22– 45)	9.7 (6.4– 13.1)
Luapula	5,552 (1,828– 8,557)	2,678 (881– 4,127)	801 (262– 1,256)	386 (126– 606)	14 (10– 19)	6.9 (4.7– 9.3)	5 (2– 7)	2.3 (0.7– 3.5)	19 (12– 26)	9.1 (5.9– 12.4)
Lusaka	11,696 (3,850– 18,024)	2,371 (780– 3,653)	1,605 (524– 2,515)	325 (106– 510)	23 (16– 32)	4.7 (3.2– 6.4)	6 (2– 9)	1.2 (0.4– 1.8)	29 (19– 39)	5.9 (3.9– 7.9)
Muchinga	4,355 (1,433– 6,711)	2,757 (908– 4,249)	556 (182– 872)	352 (115– 552)	17 (12– 24)	10.9 (7.5– 14.9)	3 (1– 5)	2.1 (0.7– 3.3)	21 (14– 28)	13.0 (8.7– 17.6)
North Western	4,180 (1,376– 6,442)	2,760 (908– 4,253)	493 (161– 772)	325 (106– 510)	13 (9– 18)	8.5 (5.9– 11.6)	2 (1– 3)	1.4 (0.5– 2.2)	15 (10– 20)	10.0 (6.7– 13.4)
Northern	6,826 (2,247– 10,519)	2,803 (923– 4,320)	974 (318– 1,526)	400 (131– 627)	29 (20– 39)	11.8 (8.1– 16.0)	6 (2– 10)	2.6 (0.9– 4.1)	35 (23– 47)	14.4 (9.6– 19.4)
Southern	9,261 (3,048– 14,272)	2,631 (866– 4,054)	1,145 (374– 1,795)	325 (106– 510)	26 (18– 35)	7.3 (5.0– 9.9)	5 (2– 7)	1.4 (0.4– 2.1)	30 (20– 41)	8.6 (5.8– 11.6)
Western	5,333 (1,755– 8,219)	2,795 (920– 4,308)	599 (196– 939)	314 (103– 492)	18 (12– 24)	9.3 (6.4– 12.6)	3 (1– 4)	1.4 (0.5– 2.2)	20 (14– 27)	10.7 (7.2– 14.4)

**Country** Province	**ETEC MSD Episodes**	**ETEC MSD Episodes per 100,000 U5C/year**	**ETEC-attributable MSS Cases**	**ETEC-attributable MSS Cases per 100,000 U5C/year**	**Direct ETEC Deaths**	**Direct Mortality Rate per 100,000 U5C/year**	**Indirect ETEC Deaths**	**Indirect Mortality Rate per 100,000 U5C/year**	**Total ETEC Deaths**	**Total ETEC Mortality Rate per 100,000 U5C/ year**
**Zimbabwe**	**67,279 (22,144– 103,679)**	**2,650 (872– 4,084)**	**6,072 (1,984– 9,516)**	**239 (78– 375)**	**321 (220– 437)**	**12.6 (8.7– 17.2)**	**26 (8– 40)**	**1.0 (0.3– 1.6)**	**347 (236– 469)**	**13.7 (9.3– 18.5)**
Bulawayo	2,037 (670– 3,139)	1,621 (534– 2,498)	188 (61– 294)	149 (49– 234)	4 (3– 6)	3.5 (2.4– 4.8)	0.2 (0– 0)	0.2 (0.1– 0.3)	5 (3– 6)	3.7 (2.5– 5.0)
Harare	8,277 (2,724– 12,756)	2,278 (750– 3,510)	866 (283– 1,357)	238 (78– 373)	32 (22– 43)	8.7 (6.0– 11.8)	2 (1– 4)	0.7 (0.2– 1.1)	34 (23– 46)	9.4 (6.4– 12.7)
Manicaland	9,296 (3,060– 14,326)	2,736 (901– 4,216)	930 (304– 1,458)	274 (89– 429)	48 (33– 66)	14.3 (9.8– 19.4)	5 (2– 7)	1.4 (0.5– 2.2)	53 (36– 72)	15.7 (10.7– 21.2)
Mashonaland Central	6,584 (2,167– 10,146)	2,879 (947– 4,436)	516 (169– 809)	226 (74– 353)	32 (22– 44)	14.1 (9.7– 19.1)	2 (1– 3)	1.0 (0.3– 1.5)	34 (23– 47)	15.0 (10.3– 20.3)
Mashonaland East	8,352 (2,749– 12,871)	2,752 (906– 4,240)	720 (235– 1,128)	237 (77– 371)	37 (26– 51)	12.4 (8.5– 16.8)	3 (1– 5)	1.0 (0.3– 1.5)	40 (28– 55)	13.3 (9.1– 18.0)
Mashonaland West	7,949 (2,616– 12,249)	2,804 (923– 4,321)	748 (245– 1,173)	264 (86– 414)	42 (29– 57)	14.9 (10.2– 20.3)	4 (1– 6)	1.4 (0.4– 2.1)	46 (31– 62)	16.3 (11.1– 22.0)
Masvingo	8,137 (2,678– 12,539)	2,804 (923– 4,321)	654 (214– 1,026)	226 (74– 353)	39 (27– 54)	13.6 (9.3– 18.5)	3 (1– 4)	0.9 (0.3– 1.4)	42 (29– 57)	14.5 (9.9– 19.7)
Matabeleland North	4,261 (1,402– 6,566)	2,844 (936– 4,383)	288 (94– 451)	192 (63– 301)	12 (9– 17)	8.3 (5.7– 11.2)	1 (0– 1)	0.5 (0.2– 0.7)	13 (9– 18)	8.7 (6.0– 11.8)
Matabeleland South	3,558 (1,171– 5,484)	2,696 (887– 4,155)	348 (114– 546)	264 (86– 414)	13 (9– 18)	9.8 (6.8– 13.4)	1 (0– 2)	0.9 (0.3– 1.4)	14 (10– 19)	10.7 (7.3– 14.5)
Midlands	8,827 (2,905– 13,603)	2,738 (901– 4,220)	814 (266– 1,276)	253 (82– 396)	60 (41– 81)	18.6 (12.7– 25.3)	4 (1– 7)	1.4 (0.5– 2.1)	64 (44– 87)	19.9 (13.6– 27.0)

Supplementary Table 5. National and subnational Shigella morbidity and mortality estimates for under-five children in 2016 for 11 eastern and central African countries.

National estimates are shown in bold. Subnational estimates are in plain text. Estimates are followed by their uncertainty intervals, shownin parentheses. Averages of regional estimates are approximately equal to national estimates (see *Methods*). DRC: Democratic Republic of Congo; MSD: moderate-to-severe diarrhoea; MSS: moderate and severe stunting; U5C: under-five child.

**Country** Province	***Shigella* MSD Episodes**	***Shigella* MSD Episodes per 100,000 U5C/year**	***Shigella* - attributable MSS Cases**	***Shigella*- attributable MSS Cases per 100,000 U5C/year**	**Direct *Shigella* Deaths**	**Direct Mortality Rate per 100,000 U5C/year**	**Indirect *Shigella* Deaths**	**Indirect Mortality Rate per 100,000 U5C/year**	**Total *Shigella* Deaths**	**Total *Shigella* Mortality Rate per 100,000 U5C/ year**
**Burundi**	**58,728 (19,753– 88,521)**	**2,802 (942– 4,223)**	**11,016 (3,612– 16,821)**	**526 (172– 802)**	**431 (313– 567)**	**20.6 (14.9– 27.1)**	**107 (36– 163)**	**5.1 (1.7– 7.8)**	**538 (375– 703)**	**25.7 (17.9– 33.5)**
Bubanza	2,504 (842– 3,774)	2,877 (968– 4,336)	462 (151– 705)	530 (174– 810)	16 (12– 22)	18.9 (13.7– 24.9)	4 (1– 6)	4.7 (1.6– 7.1)	21 (14– 27)	23.6 (16.5– 30.8)
Bujumbura Mairie	2,395 (805– 3,609)	2,606 (876– 3,928)	288 (95– 440)	314 (103– 479)	12 (9– 16)	13.0 (9.5– 17.2)	2 (1– 2)	1.7 (0.5– 2.5)	14 (10– 18)	14.7 (10.5– 19.2)
Bujumbura Rural	4,912 (1,652– 7,404)	2,819 (948– 4,249)	913 (300– 1,393)	524 (172– 800)	38 (28– 50)	21.8 (15.8– 28.7)	7 (2– 11)	4.0 (1.3– 6.1)	45 (32– 58)	25.8 (18.2– 33.6)
Bururi	2,451 (825– 3,695)	2,695 (906– 4,062)	451 (148– 688)	496 (163– 757)	17 (12– 22)	18.4 (13.4– 24.3)	3 (1– 5)	3.3 (1.1– 5.0)	20 (14– 26)	21.7 (15.4– 28.3)
Cankuzo	1,664 (560– 2,508)	2,858 (961– 4,308)	332 (109– 508)	571 (187– 872)	12 (9– 15)	20.1 (14.6– 26.5)	3 (1– 5)	5.6 (1.9– 8.5)	15 (10– 20)	25.7 (17.8– 33.6)
Cibitoke	3,292 (1,107– 4,962)	2,783 (936– 4,195)	660 (216– 1,008)	558 (183– 852)	19 (14– 25)	16.1 (11.7– 21.2)	6 (2– 10)	5.4 (1.8– 8.2)	25 (17– 33)	21.5 (14.7– 28.2)
Gitega	5,705 (1,919– 8,599)	2,820 (949– 4,251)	979 (321– 1,497)	484 (159– 740)	37 (27– 48)	18.2 (13.2– 24.0)	10 (3– 15)	4.8 (1.6– 7.3)	47 (32– 61)	23.0 (16.0– 30.1)
Karuzi	2,866 (964– 4,320)	2,590 (871– 3,904)	647 (212– 988)	585 (192– 893)	24 (17– 31)	21.3 (15.5– 28.0)	6 (2– 9)	5.4 (1.8– 8.2)	29 (21– 39)	26.6 (18.6– 34.8)
Kayanza	4,196 (1,411– 6,324)	2,792 (939– 4,209)	910 (298– 1,389)	605 (199– 924)	32 (23– 42)	21.1 (15.3– 27.7)	8 (3– 12)	5.4 (1.8– 8.2)	40 (28– 52)	26.5 (18.5– 34.6)
Kirundo	4,604 (1,549– 6,940)	2,948 (992– 4,444)	872 (286– 1,331)	558 (183– 852)	37 (27– 49)	24.0 (17.5– 31.6)	13 (4– 19)	8.1 (2.7– 12.3)	50 (34– 66)	32.1 (22.0– 42.1)
Makamba	2,358 (793– 3,555)	2,656 (894– 4,004)	409 (134– 625)	461 (151– 704)	15 (11– 19)	16.6 (12.1– 21.9)	2 (1– 4)	2.8 (0.9– 4.2)	17 (12– 22)	19.4 (13.8– 25.3)
Muramvya	2,005 (674– 3,022)	2,639 (887– 3,977)	434 (142– 663)	571 (187– 872)	20 (14– 26)	26.2 (19.0– 34.4)	5 (2– 7)	6.0 (2.0– 9.1)	24 (17– 32)	32.2 (22.5– 42.0)
Muyinga	4,874 (1,639– 7,347)	2,901 (976– 4,373)	867 (284– 1,325)	516 (169– 789)	37 (27– 49)	22.3 (16.2– 29.3)	12 (4– 17)	6.9 (2.3– 10.4)	49 (34– 64)	29.1 (20.0– 38.1)

**Country** Province	***Shigella* MSD Episodes**	***Shigella* MSD Episodes per 100,000 U5C/year**	***Shigella* -attributable MSS Cases**	***Shigella*-attributable MSS Cases per 100,000 U5C/year**	**Direct *Shigella* Deaths**	**Direct Mortality Rate per 100,000 U5C/year**	**Indirect *Shigella* Deaths**	**Indirect Mortality Rate per 100,000 U5C/year**	**Total *Shigella* Deaths**	**Total *Shigella* Mortality Rate per 100,000 U5C/ year**
Mwaro	2,023 (680– 3,049)	2,691 (905– 4,056)	393 (129– 601)	523 (172– 799)	19 (14– 24)	24.8 (18.0– 32.6)	3 (1– 5)	4.2 (1.4– 6.4)	22 (15– 28)	29.0 (20.5– 37.7)
Ngozi	5,168 (1,738– 7,789)	2,932 (986– 4,419)	984 (322– 1,503)	558 (183– 852)	41 (30– 54)	23.3 (16.9– 30.6)	12 (4– 18)	6.9 (2.3– 10.4)	53 (37– 69)	30.1 (20.8– 39.4)
Rumonge	2,087 (702– 3,145)	2,729 (918– 4,113)	423 (139– 647)	554 (182– 845)	15 (11– 20)	20.2 (14.7– 26.6)	4 (1– 6)	4.8 (1.6– 7.3)	19 (13– 25)	25.1 (17.5– 32.7)
Rutana	2,568 (864– 3,871)	2,875 (967– 4,333)	445 (146– 679)	498 (163– 760)	19 (14– 25)	21.5 (15.6– 28.3)	4 (1– 5)	4.0 (1.3– 6.1)	23 (16– 30)	25.5 (18.0– 33.2)
Ruyigi	3,056 (1,028– 4,607)	2,882 (969– 4,343)	547 (179– 835)	516 (169– 787)	21 (15– 28)	20.0 (14.5– 26.3)	4 (1– 7)	4.2 (1.4– 6.4)	26 (18– 33)	24.2 (17.0– 31.5)
**DRC**	**397,229 (133,608– 598,744)**	**2,823 (950– 4,255)**	**68,418 (22,473– 104,495)**	**486 (160– 743)**	**2,161 (1,572– 2,844)**	**15.4 (11.2– 20.2)**	**689 (228– 1,045)**	**4.9 (1.6– 7.4)**	**2,851 (1,957– 3,734)**	**20.3 (13.9– 26.5)**
Bandundu	43,774 (14,723– 65,980)	2,903 (976– 4,376)	7,230 (2,375– 11,043)	480 (158– 732)	214 (155– 281)	14.2 (10.3– 18.7)	58 (19– 88)	3.8 (1.3– 5.8)	272 (189– 355)	18.0 (12.5– 23.5)
Bas Congo	16,004 (5,383– 24,123)	2,839 (955– 4,280)	3,073 (1,009– 4,693)	545 (179– 832)	72 (52– 94)	12.7 (9.2– 16.7)	31 (10– 47)	5.5 (1.8– 8.4)	103 (68– 135)	18.2 (12.2– 24.0)
Equateur	41,631 (14,002– 62,750)	2,967 (998– 4,473)	6,331 (2,080– 9,665)	451 (148– 689)	250 (182– 329)	17.8 (13.0– 23.5)	74 (24– 112)	5.3 (1.7– 8.0)	324 (224– 424)	23.1 (16.0– 30.2)
Kasai Occidental	33,811 (11,372– 50,964)	2,982 (1,003– 4,495)	6,797 (2,231– 10,376)	599 (197– 915)	194 (141– 255)	17.1 (12.4– 22.5)	85 (28– 129)	7.5 (2.5– 11.4)	279 (186– 368)	24.6 (16.4– 32.5)
Kasai Oriental	43,554 (14,649– 65,649)	2,925 (984– 4,409)	7,797 (2,560– 11,904)	524 (172– 800)	262 (191– 345)	17.6 (12.8– 23.2)	84 (28– 127)	5.6 (1.9– 8.5)	346 (237– 453)	23.2 (15.9– 30.4)
Katanga	51,051 (17,171– 76,949)	2,867 (964– 4,321)	9,212 (3,025– 14,068)	517 (170– 790)	281 (205– 370)	15.8 (11.5– 20.8)	95 (31– 144)	5.3 (1.8– 8.1)	377 (257– 494)	21.1 (14.5– 27.7)
Kinshasa	36,980 (12,438– 55,739)	2,554 (859– 3,849)	2,962 (973– 4,518)	205 (67– 312)	104 (75– 137)	7.2 (5.2– 9.4)	10 (3– 15)	0.7 (0.2– 1.1)	114 (82– 149)	7.9 (5.6– 10.3)
Maniema	10,200 (3,431– 15,374)	2,730 (918– 4,115)	1,728 (568– 2,640)	463 (152– 706)	58 (42– 77)	15.6 (11.3– 20.5)	15 (5– 23)	4.0 (1.3– 6.0)	73 (51– 96)	19.6 (13.6– 25.6)
Nord Kivu	25,852 (8,695– 38,967)	2,678 (901– 4,037)	5,432 (1,784– 8,295)	563 (185– 859)	138 (100– 181)	14.3 (10.4– 18.8)	41 (13– 62)	4.2 (1.4– 6.4)	179 (123– 234)	18.5 (12.8– 24.2)
Orientale	74,370 (25,014– 112,097)	2,756 (927– 4,154)	13,690 (4,497– 20,909)	507 (167– 775)	457 (333– 602)	16.9 (12.3– 22.3)	141 (46– 213)	5.2 (1.7– 7.9)	598 (412– 783)	22.2 (15.3– 29.0)

**Country** Province	***Shigella* MSD Episodes**	***Shigella* MSD Episodes per 100,000 U5C/year**	***Shigella* -attributable MSS Cases**	***Shigella*-attributable MSS Cases per 100,000 U5C/year**	**Direct *Shigella* Deaths**	**Direct Mortality Rate per 100,000 U5C/year**	**Indirect *Shigella* Deaths**	**Indirect Mortality Rate per 100,000 U5C/year**	**Total *Shigella* Deaths**	**Total *Shigella* Mortality Rate per 100,000 U5C/ year**
Sud Kivu	20,003 (6,728– 30,151)	2,830 (952– 4,266)	4,166 (1,368– 6,361)	589 (194– 900)	131 (95– 172)	18.5 (13.5– 24.4)	56 (18– 84)	7.9 (2.6– 11.9)	186 (125– 245)	26.4 (17.7– 34.7)
**Ethiopia**	**419,014 (140,935– 631,579)**	**2,834 (953– 4,272)**	**66,478 (21,837– 101,541)**	**450 (148– 687)**	**1,248 (907– 1,642)**	**8.4 (6.1– 11.1)**	**328 (108– 497)**	**2.2 (0.7– 3.4)**	**1,576 (1,096– 2,059)**	**10.7 (7.4– 13.9)**
Addis Ababa	13,040 (4,386– 19,655)	2,021 (680– 3,047)	1,203 (395– 1,835)	187 (61– 285)	10 (7– 14)	1.6 (1.2– 2.1)	1 (0– 2)	0.2 (0.1– 0.3)	12 (8– 15)	1.8 (1.3– 2.4)
Afar	8,578 (2,885– 12,930)	2,859 (962– 4,310)	1,522 (500– 2,324)	507 (167– 775)	34 (25– 45)	11.5 (8.4– 15.1)	11 (4– 17)	3.8 (1.3– 5.7)	46 (31– 60)	15.3 (10.5– 20.0)
Amhara	108,689 (36,558– 163,827)	2,889 (972– 4,355)	18,739 (6,150– 28,605)	498 (163– 760)	371 (269– 488)	9.8 (7.2– 13.0)	98 (32– 149)	2.6 (0.9– 3.9)	469 (326– 612)	12.5 (8.7– 16.3)
Benishangul -Gumuz	4,300 (1,446– 6,482)	2,822 (949– 4,254)	773 (254– 1,180)	507 (167– 775)	10 (8– 14)	6.9 (5.0– 9.1)	4 (1– 6)	2.7 (0.9– 4.1)	15 (10– 19)	9.6 (6.5– 12.6)
Dire Dawa	2,095 (705– 3,157)	2,511 (845– 3,785)	408 (134– 624)	490 (161– 748)	5 (3– 6)	5.6 (4.1– 7.4)	2 (1– 3)	2.2 (0.7– 3.4)	7 (4– 9)	7.8 (5.3– 10.3)
Gambela	1,722 (579– 2,595)	2,757 (927– 4,155)	196 (64– 299)	314 (103– 479)	4 (3– 5)	6.6 (4.8– 8.7)	1 (0– 1)	1.2 (0.4– 1.9)	5 (3– 6)	7.9 (5.6– 10.3)
Harari	1,120 (377– 1,688)	2,633 (886– 3,969)	176 (58– 269)	414 (136– 632)	2 (2– 3)	5.6 (4.1– 7.4)	1 (0– 1)	1.5 (0.5– 2.3)	3 (2– 4)	7.1 (5.0– 9.3)
Oromia	150,169 (50,509– 226,350)	2,887 (971– 4,352)	22,860 (7,510– 34,901)	440 (144– 671)	479 (348– 630)	9.2 (6.7– 12.1)	112 (37– 170)	2.2 (0.7– 3.3)	591 (413– 771)	11.4 (7.9– 14.8)
SNNPR	82,353 (27,699– 124,130)	2,886 (971– 4,350)	14,104 (4,633– 21,539)	494 (162– 755)	214 (156– 282)	7.5 (5.5– 9.9)	73 (24– 111)	2.6 (0.9– 3.9)	288 (197– 378)	10.1 (6.9– 13.2)
Somali	23,026 (7,745– 34,706)	2,839 (955– 4,280)	3,034 (997– 4,630)	374 (123– 571)	63 (46– 83)	7.8 (5.7– 10.3)	14 (5– 22)	1.8 (0.6– 2.7)	78 (54– 101)	9.6 (6.7– 12.5)
Tigray	23,922 (8,046– 36,058)	2,752 (925– 4,147)	3,463 (1,136– 5,286)	398 (131– 608)	54 (40– 72)	6.3 (4.6– 8.2)	10 (3– 15)	1.1 (0.4– 1.7)	64 (45– 84)	7.4 (5.2– 9.6)
**Kenya**	**207,186 (69,687– 312,291)**	**2,895 (974– 4,364)**	**22,267 (7,315– 33,983)**	**311 (102– 475)**	**537 (390– 706)**	**7.5 (5.5– 9.9)**	**68 (22– 102)**	**0.9 (0.3– 1.4)**	**604 (431– 789)**	**8.4 (6.0– 11.0)**
Central	20,205 (6,796– 30,454)	2,655 (893– 4,001)	1,736 (570– 2,648)	228 (75– 348)	41 (30– 54)	5.4 (3.9– 7.1)	4 (1– 6)	0.5 (0.2– 0.7)	45 (32– 58)	5.9 (4.2– 7.7)
Coast	17,162	2,952	2,132	367	41	7.0	7	1.2	48	8.2

**Country** Province	***Shigella* MSD Episodes**	***Shigella* MSD Episodes per 100,000 U5C/year**	***Shigella* - attributable MSS Cases**	***Shigella*-attributable MSS Cases per 100,000 U5C/year**	**Direct *Shigella* Deaths**	**Direct Mortality Rate per 100,000 U5C/year**	**Indirect *Shigella* Deaths**	**Indirect Mortality Rate per 100,000 U5C/year**	**Total *Shigella* Deaths**	**Total *Shigella* Mortality Rate per 100,000 U5C/ year**
	(5,772– 25,868)	(993– 4,449)	(700– 3,255)	(120– 560)	(30– 54)	(5.1– 9.2)	(2– 10)	(0.4– 1.8)	(34– 62)	(5.8– 10.7)
Eastern	29,425	2,959	3,384	340	89	9.0	9	0.9	98	9.9
	(9,897– 44,352)	(995– 4,461)	(1,112– 5,168)	(112– 520)	(65– 117)	(6.5– 11.8)	(3– 14)	(0.3– 1.4)	(70– 128)	(7.1– 12.9)
Nairobi	12,567	2,472	1,095	215	22	4.3	3	0.5	25	4.8
	(4,227– 18,943)	(831– 3,725)	(360– 1,670)	(71– 329)	(16– 29)	(3.1– 5.7)	(1– 4)	(0.2– 0.8)	(18– 32)	(3.5– 6.3)
North	12,013	3,040	1,252	317	34	8.7	4	1.1	39	9.8
Eastern	(4,041– 18,107)	(1,023– 4,583)	(411– 1,910)	(104– 483)	(25– 45)	(6.3– 11.4)	(1– 7)	(0.4– 1.7)	(28– 51)	(7.0– 12.8)
Nyanza	33,425	2,979	3,313	295	80	7.1	12	1.1	93	8.2
	(11,242– 50,381)	(1,002– 4,490)	(1,088– 5,055)	(97– 451)	(58– 105)	(5.2– 9.4)	(4– 19)	(0.4– 1.7)	(66– 121)	(5.9– 10.8)
Rift Valley	54,798	2,922	6,490	346	161	8.6	19	1.0	180	9.6
	(18,431– 82,597)	(983– 4,404)	(2,132– 9,907)	(114– 528)	(117– 212)	(6.3– 11.3)	(6– 29)	(0.3– 1.5)	(129– 235)	(6.9– 12.5)
Western	27,592	3,005	2,866	312	68	7.4	9	1.0	77	8.4
	(9,280– 41,589)	(1,011– 4,530)	(941– 4,373)	(103– 476)	(50– 90)	(5.4– 9.8)	(3– 14)	(0.3– 1.5)	(55– 101)	(6.0– 11.0)
**Malawi**	**83,314**	**2,825**	**11,241**	**381**	**243**	**8.2**	**48**	**1.6**	**290**	**9.8**
	**(28,023– 125,579)**	**(950– 4,259)**	**(3,692– 17,166)**	**(125– 582)**	**(176– 319)**	**(6.0– 10.8)**	**(16– 72)**	**(0.5– 2.5)**	**(205– 378)**	**(6.9– 12.8)**
Central	34,997	2,818	4,663	375	100	8.0	20	1.6	120	9.7
	(11,771– 52,751)	(948– 4,247)	(1,531– 7,120)	(123– 573)	(73– 131)	(5.8– 10.6)	(7– 30)	(0.5– 2.4)	(85– 156)	(6.8– 12.6)
Northern	10,150	2,811	1,333	369	35	9.6	5	1.4	40	11.0
	(3,414– 15,300)	(945– 4,237)	(438– 2,035)	(121– 564)	(25– 46)	(7.0– 12.6)	(2– 8)	(0.5– 2.1)	(28– 52)	(7.8– 14.3)
Southern	38,167	2,836	5,245	390	108	8.0	23	1.7	131	9.7
	(12,837– 57,529)	(954– 4,275)	(1,723– 8,011)	(128– 595)	(79– 142)	(5.8– 10.6)	(8– 35)	(0.6– 2.6)	(92– 171)	(6.8– 12.7)
**Mozambique**	**134,722**	**2,821**	**22,336**	**468**	**402**	**8.4**	**143**	**3.0**	**545**	**11.4**
	**(45,314– 203,067)**	**(949– 4,251)**	**(7,336– 34,111)**	**(154– 714)**	**(292– 529)**	**(6.1– 11.1)**	**(47– 216)**	**(1.0– 4.5)**	**(371– 716)**	**(7.8– 15.0)**
Cabo	10,501	2,970	1,958	554	61	17.3	18	5.1	79	22.4
Delgado	(3,532– 15,828)	(999– 4,476)	(643– 2,989)	(182– 845)	(45– 81)	(12.6– 22.8)	(6– 27)	(1.7– 7.7)	(55– 104)	(15.5– 29.4)
Gaza	6,187	2,696	721	314	9	4.0	3	1.4	12	5.4
	(2,081– 9,326)	(907– 4,063)	(237– 1,099)	(103– 479)	(7– 12)	(2.9– 5.3)	(1– 5)	(0.5– 2.1)	(8– 16)	(3.7– 7.1)
Inhambane	8,018	2,792	1,188	414	11	3.8	4	1.2	14	5.0
	(2,697– 12,085)	(939– 4,209)	(390– 1,813)	(136– 632)	(8– 14)	(2.7– 5.0)	(1– 5)	(0.4– 1.9)	(10– 19)	(3.4– 6.6)
Manica	9,853	2,721	1,638	453	18	5.0	9	2.5	27	7.5
	(3,314– 14,851)	(915– 4,102)	(538– 2,502)	(149– 691)	(13– 24)	(3.6– 6.6)	(3– 14)	(0.8– 3.8)	(18– 36)	(4.9– 10.0)
Maputo	12,763	2,392	1,487	279	24	4.5	5	1.0	29	5.5

**Country** Province	***Shigella* MSD Episodes**	***Shigella* MSD Episodes per 100,000 U5C/year**	***Shigella* -attributable MSS Cases**	***Shigella*-attributable MSS Cases per 100,000 U5C/year**	**Direct *Shigella* Deaths**	**Direct Mortality Rate per 100,000 U5C/year**	**Indirect *Shigella* Deaths**	**Indirect Mortality Rate per 100,000 U5C/year**	**Total *Shigella* Deaths**	**Total *Shigella* Mortality Rate per 100,000 U5C/ year**
	(4,293– 19,238)	(804– 3,605)	(488– 2,269)	(91– 425)	(18– 32)	(3.3– 6.0)	(2– 8)	(0.3– 1.4)	(21– 38)	(3.8– 7.2)
Nampula	26,969	2,878	5,168	552	77	8.2	25	2.7	102	10.9
	(9,071– 40,650)	(968– 4,338)	(1,697– 7,891)	(181– 842)	(56– 101)	(5.9– 10.8)	(8– 38)	(0.9– 4.1)	(70– 134)	(7.4– 14.3)
Niassa	8,624	2,805	1,610	524	11	3.5	8	2.6	19	6.1
	(2,901– 12,999)	(944– 4,229)	(528– 2,458)	(172– 800)	(8– 14)	(2.6– 4.6)	(3– 12)	(0.9– 4.0)	(12– 25)	(3.8– 8.2)
Sofala	10,749	2,799	1,639	427	19	4.9	8	2.1	27	7.0
	(3,616– 16,203)	(941– 4,219)	(538– 2,503)	(140– 652)	(14– 25)	(3.6– 6.4)	(3– 12)	(0.7– 3.2)	(18– 35)	(4.7– 9.2)
Tete	13,280	2,892	2,154	469	59	12.9	17	3.6	76	16.5
	(4,467– 20,016)	(973– 4,359)	(707– 3,288)	(154– 716)	(43– 78)	(9.4– 17.0)	(5– 25)	(1.2– 5.5)	(52– 99)	(11.4– 21.6)
Zambezia	27,779	3,010	4,773	517	113	12.3	42	4.6	156	16.9
	(9,343– 41,871)	(1,013– 4,538)	(1,568– 7,289)	(170– 790)	(82– 149)	(8.9– 16.2)	(14– 64)	(1.5– 7.0)	(105– 205)	(11.4– 22.2)
**Rwanda**	**50,574**	**2,831**	**7,606**	**426**	**103**	**5.8**	**25**	**1.4**	**128**	**7.2**
	**(17,011– 76,231)**	**(952– 4,268)**	**(2,498– 11,615)**	**(140– 650)**	**(75– 136)**	**(4.2– 7.6)**	**(8– 38)**	**(0.5– 2.1)**	**(90– 168)**	**(5.0– 9.4)**
Eastern	12,135	2,854	1,780	419	25	6.0	7	1.6	32	7.5
	(4,082– 18,291)	(960– 4,302)	(585– 2,718)	(137– 639)	(18– 33)	(4.3– 7.8)	(2– 10)	(0.5– 2.4)	(22– 42)	(5.2– 9.8)
Kigali City	4,994	2,712	517	281	8	4.5	1	0.4	9	5.0
	(1,680– 7,528)	(912– 4,088)	(170– 789)	(92– 429)	(6– 11)	(3.3– 6.0)	(0– 1)	(0.1– 0.7)	(7– 12)	(3.6– 6.5)
Northern	7,742	2,768	1,221	437	14	5.1	4	1.3	18	6.4
	(2,604– 11,670)	(931– 4,173)	(401– 1,865)	(143– 667)	(10– 19)	(3.7– 6.7)	(1– 6)	(0.4– 2.0)	(12– 23)	(4.5– 8.3)
Southern	11,733	2,888	1,774	437	27	6.5	6	1.5	33	8.1
	(3,946– 17,684)	(971– 4,353)	(583– 2,710)	(143– 667)	(19– 35)	(4.7– 8.6)	(2– 9)	(0.5– 2.3)	(23– 43)	(5.6– 10.5)
Western	13,971	2,846	2,313	471	29	5.9	8	1.6	37	7.5
	(4,699– 21,058)	(957– 4,289)	(759– 3,531)	(155– 719)	(21– 38)	(4.3– 7.7)	(3– 12)	(0.5– 2.4)	(25– 48)	(5.2– 9.8)
**Tanzania**	**269,496**	**2,889**	**36,170**	**388**	**652**	**7.0**	**130**	**1.4**	**783**	**8.4**
	**(90,645– 406,212)**	**(972– 4,354)**	**(11,881– 55,246)**	**(127– 592)**	**(474– 859)**	**(5.1– 9.2)**	**(43– 197)**	**(0.5– 2.1)**	**(552– 1,020)**	**(5.9– 10.9)**
Central	33,547	2,958	3,974	350	96	8.5	12	1.1	108	9.5
	(11,283– 50,565)	(995– 4,459)	(1,305– 6,069)	(115– 535)	(70– 126)	(6.2– 11.1)	(4– 18)	(0.4– 1.6)	(77– 141)	(6.8– 12.5)
Eastern	36,036	2,717	3,920	295	78	5.9	12	0.9	90	6.8
	(12,120– 54,316)	(914– 4,095)	(1,288– 5,982)	(97– 451)	(57– 102)	(4.3– 7.7)	(4– 18)	(0.3– 1.4)	(64– 117)	(4.8– 8.8)
Lake	74,926	2,983	10,356	412	178	7.1	44	1.7	221	8.8
	(25,201– 112,936)	(1,003– 4,497)	(3,402– 15,818)	(135– 630)	(129– 234)	(5.1– 9.3)	(14– 66)	(0.6– 2.6)	(154– 289)	(6.1– 11.5)
Northern	30,686	2,687	4,873	427	69	6.1	16	1.4	85	7.5

**Country** Province	***Shigella* MSD Episodes**	***Shigella* MSD Episodes per 100,000 U5C/year**	***Shigella* - attributable MSS Cases**	***Shigella*- attributable MSS Cases per 100,000 U5C/year**	**Direct *Shigella* Deaths**	**Direct Mortality Rate per 100,000 U5C/year**	**Indirect *Shigella* Deaths**	**Indirect Mortality Rate per 100,000 U5C/year**	**Total *Shigella* Deaths**	**Total *Shigella* Mortality Rate per 100,000 U5C/ year**
	(10,321– 46,254)	(904– 4,050)	(1,601– 7,441)	(140– 652)	(50– 91)	(4.4– 8.0)	(5– 24)	(0.5– 2.1)	(60– 111)	(5.2– 9.7)
Southern	13,144	2,923	1,688	375	27	6.1	5	1.2	33	7.3
	(4,421– 19,812)	(983– 4,406)	(554– 2,578)	(123– 573)	(20– 36)	(4.4– 8.0)	(2– 8)	(0.4– 1.8)	(23– 43)	(5.1– 9.5)
S Highlands	47,026	2,950	7,098	445	114	7.2	26	1.6	140	8.8
	(15,817– 70,882)	(992– 4,446)	(2,330– 10,836)	(146– 680)	(83– 150)	(5.2– 9.4)	(9– 39)	(0.5– 2.5)	(98– 183)	(6.2– 11.5)
Western	28,660	3,023	3,600	380	79	8.3	13	1.4	92	9.7
	(9,640– 43,199)	(1,017– 4,556)	(1,183– 5,497)	(125– 580)	(57– 104)	(6.1– 11.0)	(4– 20)	(0.5– 2.1)	(65– 120)	(6.9– 12.7)
Zanzibar	5,472	2,448	661	295	11	5.0	1	0.7	13	5.6
	(1,840– 8,248)	(823– 3,690)	(217– 1,008)	(97– 451)	(8– 15)	(3.6– 6.5)	(0– 2)	(0.2– 1.0)	(9– 16)	(4.0– 7.3)
**Uganda**	**205,847**	**2,804**	**24,597**	**335**	**549**	**7.5**	**102**	**1.4**	**651**	**8.9**
	**(69,236– 310,273)**	**(943– 4,227)**	**(8,081– 37,549)**	**(110– 512)**	**(400– 723)**	**(5.4– 9.9)**	**(34– 154)**	**(0.5– 2.1)**	**(460– 849)**	**(6.3– 11.6)**
Acholi	10,206	2,854	1,096	307	31	8.7	3	0.8	34	9.6
	(3,433– 15,383)	(960– 4,302)	(360– 1,674)	(101– 468)	(23– 41)	(6.4– 11.5)	(1– 5)	(0.3– 1.3)	(25– 45)	(6.9– 12.5)
Ankole	14,479	2,944	1,804	367	51	10.5	9	1.7	60	12.2
	(4,870– 21,824)	(990– 4,437)	(593– 2,754)	(120– 560)	(37– 68)	(7.6– 13.8)	(3– 13)	(0.6– 2.6)	(43– 78)	(8.6– 15.9)
Bugishu	7,616	2,802	1,121	412	27	9.9	5	1.9	32	11.8
	(2,561– 11,479)	(942– 4,224)	(368– 1,712)	(135– 630)	(20– 35)	(7.2– 13.0)	(2– 8)	(0.6– 2.9)	(23– 42)	(8.3– 15.3)
Bukedi	9,734	2,804	965	278	22	6.4	4	1.2	27	7.6
	(3,274– 14,672)	(943– 4,226)	(317– 1,472)	(91– 424)	(16– 29)	(4.7– 8.5)	(1– 6)	(0.4– 1.8)	(19– 35)	(5.4– 10.0)
Bunyoro	13,672	2,855	1,975	412	34	7.2	10	2.0	44	9.1
	(4,599– 20,608)	(960– 4,304)	(649– 3,016)	(135– 630)	(25– 45)	(5.2– 9.4)	(3– 14)	(0.7– 3.0)	(30– 57)	(6.3– 12.0)
Busoga	22,335	2,788	2,815	351	52	6.4	13	1.6	64	8.0
	(7,512– 33,665)	(938– 4,203)	(925– 4,296)	(115– 536)	(38– 68)	(4.7– 8.5)	(4– 19)	(0.5– 2.4)	(45– 84)	(5.6– 10.5)
Central 1	23,875	2,753	2,459	284	61	7.1	6	0.7	67	7.7
	(8,030– 35,987)	(926– 4,149)	(808– 3,754)	(93– 433)	(45– 81)	(5.1– 9.3)	(2– 9)	(0.2– 1.0)	(48– 88)	(5.5– 10.1)
Central 2	21,538	2,827	2,494	327	55	7.2	9	1.2	64	8.4
	(7,244– 32,464)	(951– 4,262)	(819– 3,807)	(108– 500)	(40– 73)	(5.3– 9.5)	(3– 14)	(0.4– 1.8)	(45– 84)	(6.0– 11.0)
Kampala	12,027	2,460	1,271	260	33	6.7	5	1.1	38	7.8
	(4,045– 18,128)	(827– 3,707)	(418– 1,939)	(85– 396)	(24– 43)	(4.8– 8.8)	(2– 8)	(0.4– 1.7)	(27– 50)	(5.5– 10.1)
Karamoja	5,908	2,839	888	427	12	5.9	5	2.4	17	8.3
	(1,987– 8,905)	(955– 4,279)	(292– 1,356)	(140– 652)	(9– 16)	(4.3– 7.8)	(2– 7)	(0.8– 3.6)	(12– 23)	(5.6– 10.9)
Kigezi	9,692	2,871	1,187	351	20	6.0	5	1.3	25	7.3
	(3,260– 14,609)	(966– 4,327)	(390– 1,811)	(115– 536)	(15– 27)	(4.4– 7.9)	(1– 7)	(0.4– 2.0)	(17– 32)	(5.1– 9.6)

**Country** Province	***Shigella* MSD Episodes**	***Shigella* MSD Episodes per 100,000 U5C/year**	***Shigella* - attributable MSS Cases**	***Shigella*- attributable MSS Cases per 100,000 U5C/year**	**Direct *Shigella* Deaths**	**Direct Mortality Rate per 100,000 U5C/year**	**Indirect *Shigella* Deaths**	**Indirect Mortality Rate per 100,000 U5C/year**	**Total *Shigella* Deaths**	**Total *Shigella* Mortality Rate per 100,000 U5C/ year**
Lango	9,432 (3,173– 14,217)	2,841 (956– 4,282)	925 (304– 1,412)	279 (91– 425)	29 (21– 39)	8.9 (6.4– 11.7)	3 (1– 5)	1.0 (0.3– 1.5)	33 (23– 43)	9.9 (7.1– 12.9)
Teso	10,215 (3,436– 15,397)	2,788 (938– 4,202)	620 (204– 945)	169 (56– 258)	28 (20– 37)	7.7 (5.6– 10.1)	2 (1– 3)	0.5 (0.2– 0.7)	30 (21– 39)	8.2 (5.9– 10.7)
Tooro	17,418 (5,859– 26,255)	2,866 (964– 4,320)	2,558 (840– 3,905)	421 (138– 643)	43 (32– 57)	7.2 (5.2– 9.4)	11 (4– 17)	1.9 (0.6– 2.9)	55 (38– 72)	9.0 (6.3– 11.8)
West Nile	17,701 (5,954– 26,680)	2,846 (957– 4,290)	2,421 (795– 3,696)	389 (128– 594)	49 (36– 65)	7.9 (5.7– 10.4)	12 (4– 19)	2.0 (0.7– 3.0)	61 (43– 80)	9.9 (6.9– 12.9)
**Zambia**	**80,631 (27,120– 121,535)**	**2,788 (938– 4,203)**	**13,072 (4,293– 19,963)**	**452 (148– 690)**	**237 (173– 312)**	**8.2 (6.0– 10.8)**	**62 (21– 95)**	**2.2 (0.7– 3.3)**	**300 (209– 392)**	**10.4 (7.2– 13.5)**
Central	8,492 (2,856– 12,801)	2,827 (951– 4,262)	1,490 (489– 2,274)	496 (163– 757)	27 (20– 36)	9.0 (6.6– 11.8)	8 (3– 12)	2.6 (0.8– 3.9)	35 (24– 45)	11.6 (8.0– 15.1)
Copperbelt	10,689 (3,595– 16,111)	2,357 (793– 3,553)	1,870 (614– 2,856)	412 (135– 630)	29 (21– 39)	6.5 (4.7– 8.5)	6 (2– 10)	1.4 (0.5– 2.1)	36 (25– 47)	7.9 (5.5– 10.3)
Eastern	9,841 (3,310– 14,833)	2,881 (969– 4,342)	1,609 (528– 2,457)	471 (155– 719)	28 (21– 37)	8.3 (6.0– 10.8)	10 (3– 15)	2.8 (0.9– 4.3)	38 (26– 50)	11.1 (7.6– 14.6)
Luapula	6,071 (2,042– 9,150)	2,928 (985– 4,413)	1,052 (345– 1,606)	507 (167– 775)	16 (11– 20)	7.5 (5.5– 9.8)	6 (2– 9)	3.0 (1.0– 4.5)	22 (15– 29)	10.5 (7.0– 13.8)
Lusaka	12,788 (4,301– 19,275)	2,592 (872– 3,907)	2,105 (692– 3,215)	427 (140– 652)	25 (18– 33)	5.2 (3.8– 6.8)	7 (2– 11)	1.5 (0.5– 2.3)	33 (23– 43)	6.7 (4.6– 8.7)
Muchinga	4,761 (1,601– 7,176)	3,015 (1,014– 4,544)	731 (240– 1,116)	463 (152– 706)	19 (14– 25)	12.0 (8.7– 15.7)	4 (1– 7)	2.7 (0.9– 4.1)	23 (16– 30)	14.7 (10.3– 19.2)
North Western	4,570 (1,537– 6,889)	3,018 (1,015– 4,548)	646 (212– 987)	427 (140– 652)	14 (10– 19)	9.3 (6.8– 12.3)	3 (1– 4)	1.9 (0.6– 2.8)	17 (12– 22)	11.2 (7.9– 14.6)
Northern	7,463 (2,510– 11,249)	3,065 (1,031– 4,619)	1,280 (420– 1,955)	526 (173– 803)	31 (23– 41)	12.9 (9.4– 16.9)	8 (3– 13)	3.4 (1.1– 5.2)	40 (28– 52)	16.3 (11.3– 21.3)
Southern	10,126 (3,406– 15,263)	2,876 (967– 4,335)	1,502 (493– 2,294)	427 (140– 652)	28 (20– 37)	7.9 (5.8– 10.4)	6 (2– 9)	1.8 (0.6– 2.7)	34 (24– 45)	9.7 (6.8– 12.7)
Western	5,831 (1,961– 8,789)	3,056 (1,028– 4,607)	787 (258– 1,202)	412 (135– 630)	19 (14– 25)	10.1 (7.4– 13.3)	3 (1– 5)	1.8 (0.6– 2.8)	23 (16– 30)	12.0 (8.5– 15.6)
**Zimbabwe**	**73,558 (24,741– 110,874)**	**2,897 (974– 4,367)**	**7,956 (2,614– 12,144)**	**313 (103– 478)**	**351 (255– 462)**	**13.8 (10.1– 18.2)**	**33 (11– 50)**	**1.3 (0.4– 2.0)**	**384 (275– 502)**	**15.1 (10.8– 19.8)**

**Country** Province	***Shigella* MSD Episodes**	***Shigella* MSD Episodes per 100,000 U5C/year**	***Shigella* -attributable MSS Cases**	***Shigella*-attributable MSS Cases per 100,000 U5C/year**	**Direct *Shigella* Deaths**	**Direct Mortality Rate per 100,000 U5C/year**	**Indirect *Shigella* Deaths**	**Indirect Mortality Rate per 100,000 U5C/year**	**Total *Shigella* Deaths**	**Total *Shigella* Mortality Rate per 100,000 U5C/ year**
Bulawayo	2,227 (749– 3,357)	1,772 (596– 2,672)	245 (81– 374)	195 (64– 298)	5 (3– 6)	3.8 (2.8– 5.0)	0 (0– 0)	0.2 (0.1– 0.4)	5 (4– 7)	4.1 (2.9– 5.3)
Harare	9,050 (3,044– 13,641)	2,490 (838– 3,754)	1,134 (373– 1,731)	312 (103– 476)	34 (25– 45)	9.5 (6.9– 12.5)	3 (1– 5)	0.9 (0.3– 1.4)	38 (27– 49)	10.4 (7.4– 13.6)
Manicaland	10,164 (3,419– 15,320)	2,991 (1,006– 4,509)	1,220 (401– 1,863)	359 (118– 548)	53 (39– 70)	15.6 (11.3– 20.5)	6 (2– 10)	1.8 (0.6– 2.8)	59 (42– 77)	17.4 (12.5– 22.8)
Mashonaland Central	7,198 (2,421– 10,850)	3,147 (1,059– 4,744)	676 (222– 1,031)	295 (97– 451)	35 (26– 46)	15.4 (11.2– 20.2)	3 (1– 4)	1.2 (0.4– 1.9)	38 (27– 50)	16.6 (11.9– 21.7)
Mashonaland East	9,132 (3,071– 13,764)	3,008 (1,012– 4,535)	943 (310– 1,440)	311 (102– 474)	41 (30– 54)	13.5 (9.8– 17.8)	4 (1– 6)	1.2 (0.4– 1.9)	45 (32– 58)	14.8 (10.6– 19.3)
Mashonaland West	8,691 (2,923– 13,099)	3,065 (1,031– 4,620)	981 (322– 1,498)	346 (114– 528)	46 (34– 61)	16.3 (11.8– 21.4)	5 (2– 8)	1.8 (0.6– 2.7)	51 (37– 67)	18.1 (12.9– 23.6)
Masvingo	8,896 (2,992– 13,409)	3,066 (1,031– 4,621)	857 (281– 1,307)	295 (97– 451)	43 (31– 57)	14.9 (10.8– 19.6)	4 (1– 5)	1.2 (0.4– 1.8)	47 (33– 61)	16.1 (11.5– 21.0)
Matabeleland North	4,658 (1,567– 7,022)	3,110 (1,046– 4,687)	377 (124– 575)	251 (83– 384)	14 (10– 18)	9.0 (6.6– 11.9)	1 (0– 1)	0.6 (0.2– 0.9)	14 (10– 19)	9.6 (6.9– 12.6)
Matabeleland South	3,891 (1,309– 5,864)	2,948 (991– 4,443)	457 (150– 697)	346 (114– 528)	14 (10– 19)	10.8 (7.8– 14.2)	2 (1– 2)	1.2 (0.4– 1.8)	16 (11– 21)	11.9 (8.5– 15.6)
Midlands	9,651 (3,246– 14,547)	2,994 (1,007– 4,513)	1,066 (350– 1,627)	331 (109– 505)	65 (48– 86)	20.3 (14.8– 26.7)	6 (2– 9)	1.8 (0.6– 2.7)	71 (51– 93)	22.1 (15.8– 28.8)

Supplementary Table 6. Distribution of risk indices for under-five children across eastern and central African countries by economic status and household setting (urban or rural).

We grouped children into wealth quintiles using DHS asset indices[1], but omitted water and sanitation assets to prevent confounding of our analysis.[2] Weighted means and upper and lower 95% confidence intervals were calculated while accounting for survey design. Abbreviations: DRC: Democratic Republic of Congo, n: number of children. Lowest to highest refer to the five wealth quintile categories.

Risk Index	Burundi	DRC	Ethiopia	Kenya	Malawi	Mozambique	Rwanda	Tanzania	Uganda	Zambia	Zimbabwe
**Urban (n)**	**596**	**2308**	**1510**	**5689**	**454**	**2904**	**734**	**1998**	**422**	**3995**	**1743**
Lowest	2.08	1.26	1.74	0.67	0.62	0.85	0.58	0.93	0.77	0.70	0.40
	(1.71, 2.45)	(1.12, 1.40)	(1.20, 2.28)	(0.62, 0.72)	(0.48, 0.76)	(0.72, 0.98)	(0.51, 0.66)	(0.79, 1.07)	(0.58, 0.96)	(0.65, 0.75)	(0.33, 0.46)
Lower	1.14	0.99	0.89	0.47	0.49	0.51	0.76	0.73	0.77	0.47	0.27
	(0.98, 1.31)	(0.89, 1.09)	(0.79, 1.00)	(0.43, 0.51)	(0.38, 0.60)	(0.44, 0.57)	(0.63, 0.90)	(0.65, 0.80)	(0.56, 0.99)	(0.43, 0.51)	(0.23, 0.31)
Middle	1.09	0.78	0.85	0.39	0.47	0.45	0.63	0.70	0.52	0.39	0.30
	(0.86, 1.33)	(0.67, 0.89)	(0.65, 1.04)	(0.37, 0.42)	(0.28, 0.66)	(0.38, 0.51)	(0.54, 0.72)	(0.63, 0.78)	(0.44, 0.59)	(0.35, 0.43)	(0.25, 0.35)
Higher	0.72	0.61	0.52	0.36	0.35	0.28	0.70	0.73	0.61	0.34	0.24
	(0.56, 0.88)	(0.54, 0.68)	(0.39, 0.65)	(0.33, 0.39)	(0.23, 0.47)	(0.25, 0.32)	(0.57, 0.84)	(0.64, 0.83)	(0.50, 0.72)	(0.31, 0.37)	(0.20, 0.27)
Highest	0.55	0.61	0.33	0.28	0.23	0.26	0.63	0.53	0.36	0.22	0.17
	(0.38, 0.72)	(0.53, 0.69)	(0.25, 0.41)	(0.24, 0.31)	(0.13, 0.32)	(0.21, 0.31)	(0.49, 0.78)	(0.47, 0.60)	(0.25, 0.47)	(0.19, 0.25)	(0.14, 0.21)
**Rural (n)**	**2780**	**5574**	**7964**	**12530**	**4052**	**6317**	**2702**	**6867**	**1625**	**7028**	**3127**
Lowest	1.97	1.66	2.64	0.92	0.73	1.29	1.05	1.38	1.06	0.94	0.56
	(1.80, 2.15)	(1.53, 1.78)	(2.47, 2.82)	(0.87, 0.97)	(0.66, 0.79)	(1.18, 1.40)	(0.97, 1.13)	(1.29, 1.47)	(0.93, 1.19)	(0.87, 1.00)	(0.51, 0.62)
Lower	1.69	1.81	2.18	0.67	0.60	1.06	0.93	1.39	1.12	0.65	0.59
	(1.56, 1.83)	(1.66, 1.96)	(2.01, 2.35)	(0.64, 0.70)	(0.54, 0.66)	(0.98, 1.15)	(0.86, 1.01)	(1.31, 1.48)	(0.97, 1.27)	(0.62, 0.69)	(0.54, 0.64)
Middle	1.30	1.58	2.15	0.63	0.60	1.10	0.87	1.29	1.26	0.61	0.58
	(1.19, 1.41)	(1.45, 1.70)	(1.94, 2.36)	(0.60, 0.67)	(0.56, 0.65)	(1.02, 1.19)	(0.80, 0.94)	(1.21, 1.37)	(1.11, 1.41)	(0.58, 0.65)	(0.52, 0.64)
Higher	1.34	1.35	1.76	0.56	0.48	0.64	0.89	1.03	1.03	0.64	0.43
	(1.21, 1.47)	(1.23, 1.47)	(1.62, 1.91)	(0.52, 0.59)	(0.44, 0.51)	(0.58, 0.70)	(0.81, 0.97)	(0.95, 1.11)	(0.88, 1.18)	(0.60, 0.68)	(0.39, 0.47)
Highest	1.07	1.33	1.48	0.50	0.48	0.51	0.79	0.83	0.76	0.53	0.33
	(0.97, 1.17)	(1.20, 1.47)	(1.36, 1.61)	(0.47, 0.53)	(0.44, 0.52)	(0.47, 0.55)	(0.71, 0.87)	(0.77, 0.88)	(0.66, 0.85)	(0.49, 0.56)	(0.29, 0.37)

References

[1]Rutstein SO, Johnson K, MEASURE OM, others. The DHS wealth index. ORC Macro, MEASURE DHS, 2004.

[2]Rheingans R, Anderson JD, Luyendijk R, Cumming O. Measuring disparities in sanitation access: does the measure matter? *Trop Med Int Health* 2014; **19**: 2–13.

Supplementary Table 7. All-cause under-five child mortality rates by province for ten years preceding the Demographic Health Survey (DHS).

We obtained these estimates from the DHS STATcompiler online tool (www.statcompiler.com).

**Country** Province	**Mortality Rate (deaths per 1,000 children)**	**Country** Province	**Mortality Rate (deaths per 1,000 children)**	**Country** Province	**Mortality Rate (deaths per 1,000 children)**	**Country** Province	**Mortality Rate (deaths per 1,000 children)**	**Country** Province	**Mortality Rate (deaths per 1,000 children)**
**Burundi (2016-17 DHS)**		**DRC (2013-14 DHS)**		**Kenya (2016 DHS)**	42 (CI: 32 – 52)	**Rwanda (2014-15 DHS)**		**Uganda (cont'd)**	
Bubanza	74 (CI: 54–95)	Bandundu	89 (CI: 75 – 102)	Central	57 (CI: 50 – 65)	Eastern	86 (CI: 76 – 97)	Tooro	81 (CI: 66 – 97)
Bujumbura Mairie	67 (CI: 34–100)	Bas Congo	124 (CI: 87 – 161)	Coast	45 (CI: 37 – 52)	Kigali City	42 (CI: 32 – 53)	West Nile	86 (CI: 69 – 103)
Bujumbura Rural	48 (CI: 34 – 62)	Equateur	132 (CI: 112 – 153)	Eastern	72 (CI: 52 – 91)	Northern	60 (CI: 51 – 70)	**Zambia (2013-14 DHS)**	
Bururi	49 (CI: 37 – 62)	Kasai Occidental	135 (CI: 93 – 177)	Nairobi	44 (CI: 33 – 55)	Southern	66 (CI: 55 – 76)	Central	80 (CI: 63 – 97)
Cankuzo	83 (CI: 64 – 103)	Kasai Oriental	122 (CI: 105 – 139)	North Eastern	82 (CI: 72 – 92)	Western	62 (CI: 52 – 72)	Copperbelt	63 (CI: 49 – 77)
Cibitoke	96 (CI: 73 – 118)	Katanga	121 (CI: 104 – 138)	Nyanza	45 (CI: 40 – 50)	**Tanzania (2014-15 DHS)**		Eastern	115 (CI: 100 – 129)
Gitega	97 (CI: 75 – 119)	Kinshasa	83 (CI: 66 – 101)	Rift Valley	64 (CI: 52 – 76)	Central	66 (CI: 53 – 80)	Luapula	98 (CI: 76 – 119)
Karuzi	61 (CI: 49 – 73)	Maniema	105 (CI: 74 – 135)	Western	42 (CI: 32 – 52)	Eastern	85 (CI: 69 – 100)	Lusaka	68 (CI: 55 – 82)
Kayanza	62 (CI: 46 – 78)	Nord Kivu	65 (CI: 51 – 79)	**Malawi (2015-16 DHS)**		Lake	88 (CI: 75 – 101)	Muchinga	88 (CI: 76 – 101)
Kirundo	145 (CI: 123 – 166)	Orientale	112 (CI: 91 – 133)	Central	42 (CI: 32 – 52)	Northern	56 (CI: 43 – 69)	North Western	66 (CI: 53 – 79)
Makamba	50 (CI: 38 – 62)	Sud Kivu	139 (CI: 116 – 161)	Northern	57 (CI: 50 – 65)	Southern	79 (CI: 56 – 102)	Northern	86 (CI: 71 – 101)
Muramvya	69 (CI: 50 – 87)	**Ethiopia (2016 DHS)**		Southern	45 (CI: 37 – 52)	S Highlands	65 (CI: 52 – 79)	Southern	68 (CI: 56 – 80)

Mwaro	43 (CI: 32 – 55)	Afar	125 (CI: 101 – 149)	**Mozambique (2011 DHS)**		Zanzibar	69 (CI: 56 – 82)	Western	73 (CI: 49 – 96)
Ngozi	109 (CI: 83 – 136)	Amhara	85 (CI: 70 – 101)	Cabo Delgado	116 (CI: 94 – 138)	**Uganda (2016 DHS)**		**Zimbabwe (2015 DHS)**	
Rumonge	71 (CI: 53 – 89)	Benishangul-Gumuz	98 (CI: 77 – 118)	Gaza	110 (CI: 88 – 133)	Acholi	69 (CI: 53 – 85)	Bulawayo	50 (CI: 34 – 66)
Rutana	64 (CI: 46 – 82)	Gambela	93 (CI: 62 – 123)	Inhambane	58 (CI: 43 – 73)	Ankole	72 (CI: 57 – 87)	Harare	58 (CI: 39 – 77)
Ruyigi	64 (CI: 47 – 81)	Harari	88 (CI: 69 – 107)	Manica	114 (CI: 96 – 133)	Bugishu	68 (CI: 54 – 81)	Manicaland	112 (CI: 78 – 147)
		Oromia	72 (CI: 52 – 93)	Maputo	96 (CI: 77 – 114)	Bukedi	72 (CI: 60 – 84)	Mashonaland Central	90 (CI: 68 – 111)
		SNNPR	79 (CI: 66 – 91)	Nampula	67 (CI: 51 – 82)	Bunyoro	89 (CI: 70 – 107)	Mashonaland East	102 (CI: 75 – 128)
		Somali	88 (CI: 69 – 108)	Niassa	101 (CI: 79 – 122)	Busoga	84 (CI: 71 – 97)	Mashonaland West	101 (CI: 64 – 137)
		Tigray	94 (CI: 80 – 108)	Sofala	105 (CI: 81 – 130)	Central 1	59 (CI: 44 – 75)	Masvingo	65 (CI: 52 – 78)
				Tete	129 (CI: 107 – 151)	Central 2	74 (CI: 57 – 91)	Matabeleland North	67 (CI: 51 – 84)
				Zambezia	142 (CI: 124 – 159)	Kampala	64 (CI: 40 – 88)	Matabeleland South	65 (CI: 40 – 91)
						Karamoja	102 (CI: 83 – 121)	Midlands	72 (CI: 56 – 89)
						Kigezi	67 (CI: 50 – 83)		
						Lango	68 (CI: 55 – 80)		
						Teso	54 (CI: 44 – 64)		
